# Recent advances in chiral NHC–gold complexes for asymmetric catalysis

**DOI:** 10.3762/bjoc.22.128

**Published:** 2026-11-05

**Authors:** Riccardo Mobili, Jules Ramacciotti, Louis Fensterbank, Virginie Mouriès-Mansuy

**Affiliations:** 1 Institut Parisien de Chimie Moléculaire, Sorbonne Université CNRS, 4 Place Jussieu, CC 229, 75252 Paris, France; 2 Laboratoire d’Activation Moléculaire, Collège de France, CNRS, Sorbonne Université, 11 Place Marcelin Berthelot, 75005 Paris, France

**Keywords:** asymmetric gold catalysis, chiral N-heterocyclic carbene (NHC) ligands, cycloisomerization reactions, ligand design

## Abstract

Homogeneous gold catalysis has emerged as a prominent area of transition-metal catalysis; however, the development of efficient asymmetric gold catalysts remains a significant challenge. Because of the linear coordination mode of gold(I), the chiral ligand is positioned opposite the substrate-binding site, complicating the generation of a chiral environment around the metal center. On the other hand, the square planar geometry of gold(III) offers a promising alternative but its redox instability has hampered its use. In recent years, chiral N-heterocyclic carbenes (NHCs) have become a valuable alternative to more established phosphine-based chiral ligands. In this review, we summarize the recent advances in the development of chiral NHC–gold complexes reported from 2019 until today. Particular emphasis is placed on ligand design principles and their impact on catalytic performance in benchmark asymmetric transformations.

## Introduction

Over the past two decades, homogeneous gold catalysis has emerged as an important area of transition-metal catalysis, providing access to molecular complexity that would otherwise be difficult to achieve under mild conditions. This reactivity arises from the ability of gold to efficiently π-activate a broad range of unsaturated hydrocarbons, such as alkynes, alkenes, and allenes, towards nucleophilic attack. Despite the remarkable advances in the field, achieving efficient asymmetric induction in gold catalysis remains a significant challenge and, in contrast to other areas of asymmetric transition metal catalysis, no single class of chiral ligands has emerged as a general solution [1–3]. Considerable efforts have therefore been devoted to this area of research. The most common oxidation states of gold are Au(I) and Au(III), and the corresponding complexes each have their own advantages and limitations in catalysis.

Gold(I) complexes are generally easy to prepare and display good stability, but their characteristic linear coordination mode places the chiral ligand opposite the substrate. This limitation is particularly relevant because stereocontrol is commonly established during an outer-sphere nucleophilic attack on the gold-activated π-system. Moreover, the low rotational barrier of the gold(I)-unsaturated substrate bond can further reduce the efficiency of asymmetric induction [4–6].

As for gold(III) complexes, they adopt a square-planar geometry, which, in some cases, can bring the chiral ligand closer to the catalytic site, thereby facilitating enantioinduction. Nevertheless, Au(III) catalysis remains underdeveloped, probably due of the high redox potential of the Au(III)/Au(I) couple, which renders Au(III) species less stable and prone to reduction to Au(I) or Au(0) species [7–9].

In this context, C-based ligands such as N-heterocyclic carbenes (NHCs) [10–11] have emerged as valuable alternatives to phosphines, owing to their strong σ-donor properties, which allow the formation of highly robust and conformationally rigid complexes. Their readily adaptable structures allow access to a wide variety of chiral NHC–gold complexes featuring different types of chirality, including central, axial, and planar chirality [1].

**Figure 1:**
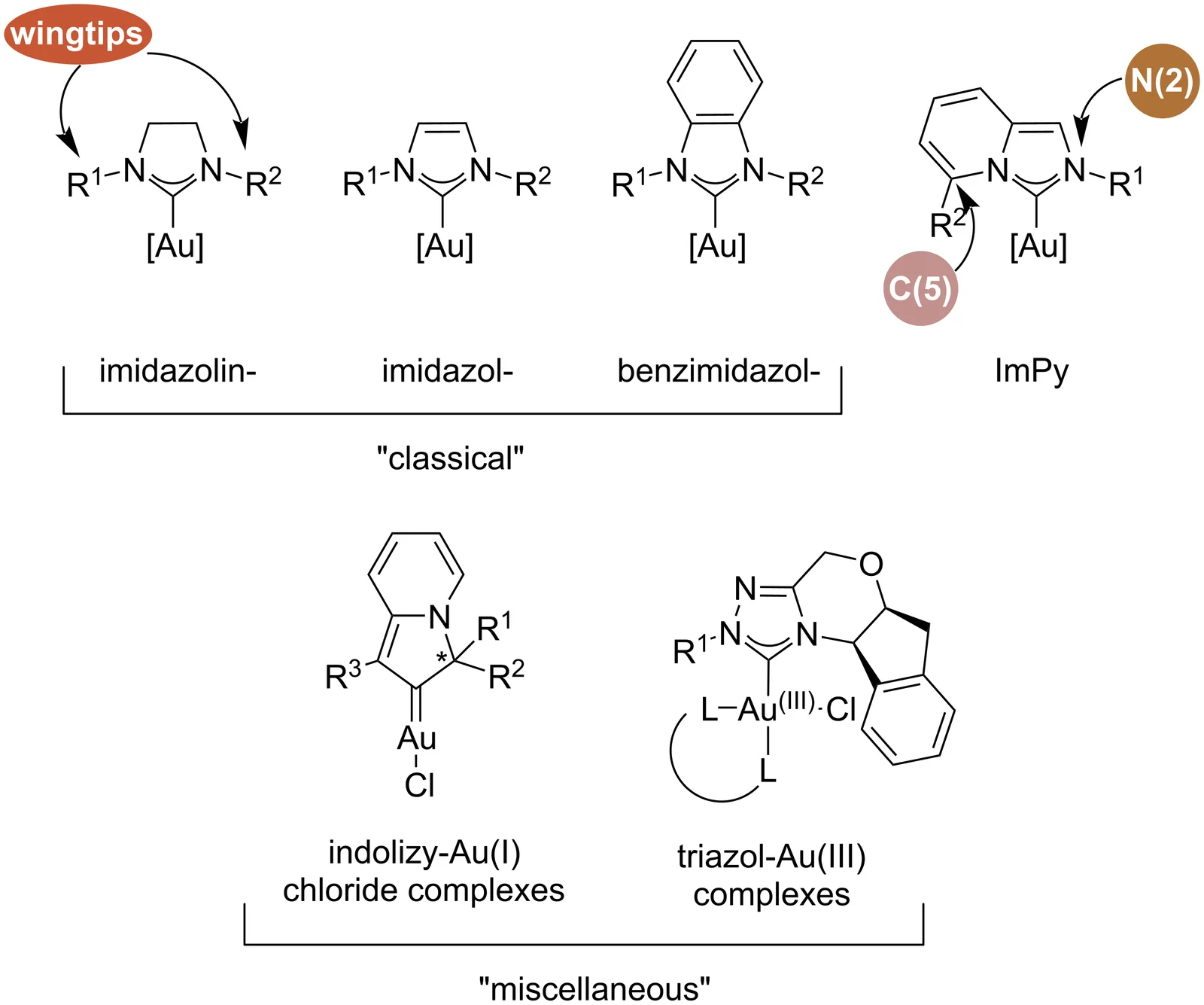
Schematic representation of the families of chiral NHC ligands described in this review.

In this review, we will focus on the application of chiral NHC–gold(I) and –gold(III) complexes in enantioselective catalysis from 2019. Since NHC is a generic term encompassing a wide variety of nitrogen-containing cyclic carbene ligands, we decided to organize this update review as follows: first, we will describe asymmetric reactions relying on chiral imidazolin-, imidazol-, and benzimidazol-2-ylidene ligands (i.e., “classical” NHCs), followed by those based on chiral imidazo[1,5-*a*]pyridin-3-ylidene (i.e., ImPy) ligands. Finally, we will discuss some chiral NHC ligands that do not belong to the carbene families described above (i.e., miscellaneous carbenes) (Figure 1).

## Review

### Chiral gold complexes based on “classical” NHC ligands

**Scheme 1:**
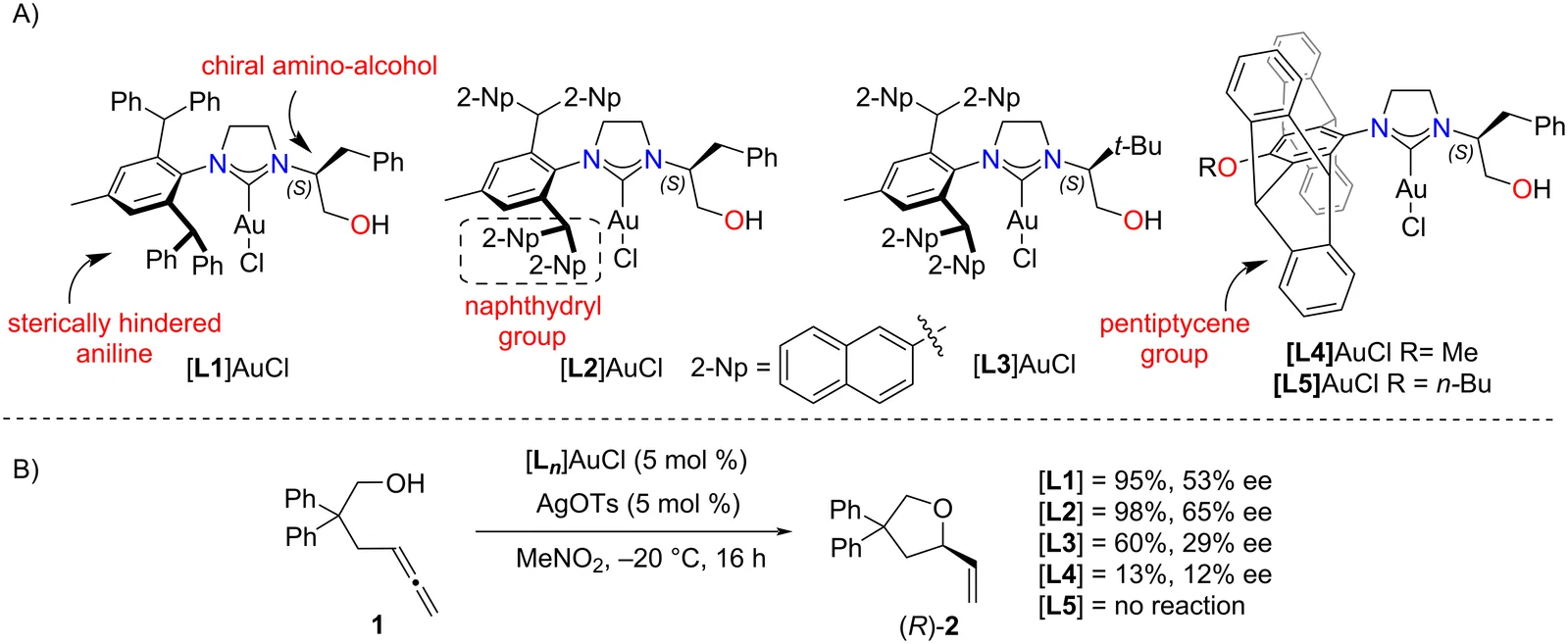
A) Structure of some chiral gold(I) complexes obtained in ref. [17]; B) cyclization of allenol **1** used as benchmark reaction to explore the catalytic performance of the same complexes.

In 2003, Herrmann et al. reported the first use of an NHC–gold(I) complex in catalysis, performing the hydration of 3-hexyne [12]. Since then, extensive research has been conducted in this field, including asymmetric catalysis, and several reviews have already covered the topic [13–16]. Focusing on the latest developments in the field, the joint work of M. Michalak and A. Poater groups is of special interest. In this collaborative study, the authors designed a series of gold(I) complexes based on chiral imidazolinium ligands obtained by reacting sterically hindered 2,6-disubstituted anilines with commercially available chiral amines or amino-alcohols (Scheme 1A) [17].

The synthesis of the NHC precursors could be achieved in four chromatography-free steps, starting from cheap building blocks. The corresponding gold(I) complexes could then be synthesized with standard procedures up to gram-scale [18–19]. The yields ranged from moderate to good when the naphthydryl derivative was present. These complexes showcased a great stability, and preserved their catalytic activity even 3 years after the synthesis.

The cyclization of allenol **1** was then studied as benchmark reaction in order to assess the asymmetric induction of these ligands (Scheme 1B) [20–24]. In this kind of reaction, the final protodeauration is presumably the limiting step [25]. For this reason, at the beginning of the optimization screening, the authors have chosen to activate the gold(I) precatalysts with AgOTs or AgOTf. These weakly basic counteranions may be useful as they can generate in situ small quantities of the related acid. The best results were obtained with AgOTs. The effect of solvent was also studied comparing the results obtained catalyzing the reaction with [**L2**]Au(I)Cl and AgOTs at room temperature. They found out that MeNO_2_ and toluene gave the best yield with 98% and 99%, respectively. However, MeNO_2_ was found to give the best enantiomeric excess (ee) with 48% compared to the 18% in toluene. By lowering the temperature to −20 °C, they were able to increase the ee up to 65%. In these optimized conditions, ligand [**L1**] gave the vinyl tetrahydrofuran derivative **2** in a yield of 95% and 53% ee. The authors further showed that increasing the steric hindrance on the aniline system by replacing phenyl rings with 2-naphthyl groups (as in [**L2**]) was beneficial for the enantioselectivity. Increasing the hindrance with a rigid pentiptycene group as in [**L4**] and [**L5**] proved instead detrimental. The benzyl moiety in beta position to the alcohol gave the best results, while a *tert*-butyl group (i.e., [**L3**]) dropped both the yield and ee to 60% and 29%, respectively. The importance of hydrogen bonding was demonstrated through an experiment in which the alcohol in [**L2**] was protected by a *tert*-butyldimethylsilyl (TBS) moiety or acetylated. In the first case, a loss of ee from 65% to 24% was observed. In the second case the yield decreased to 73% and the ee dropped to 16%. This confirmed the crucial role of the alcohol in the enantiodiscrimination and supported their initial hypothesis. These results indicate a dependence on both the steric hindrance and conformation flexibility of the NHC wingtips. In summary, merging one highly hindered but flexible wingtip with a moderately hindered chiral primary alcohol on a NHC ligand was found to be the optimal design strategy for a high-performance gold(I) catalyst that delivers the tetrahydrofuran derivative **2** in excellent yield and good enantiomeric excesses.

**Scheme 2:**
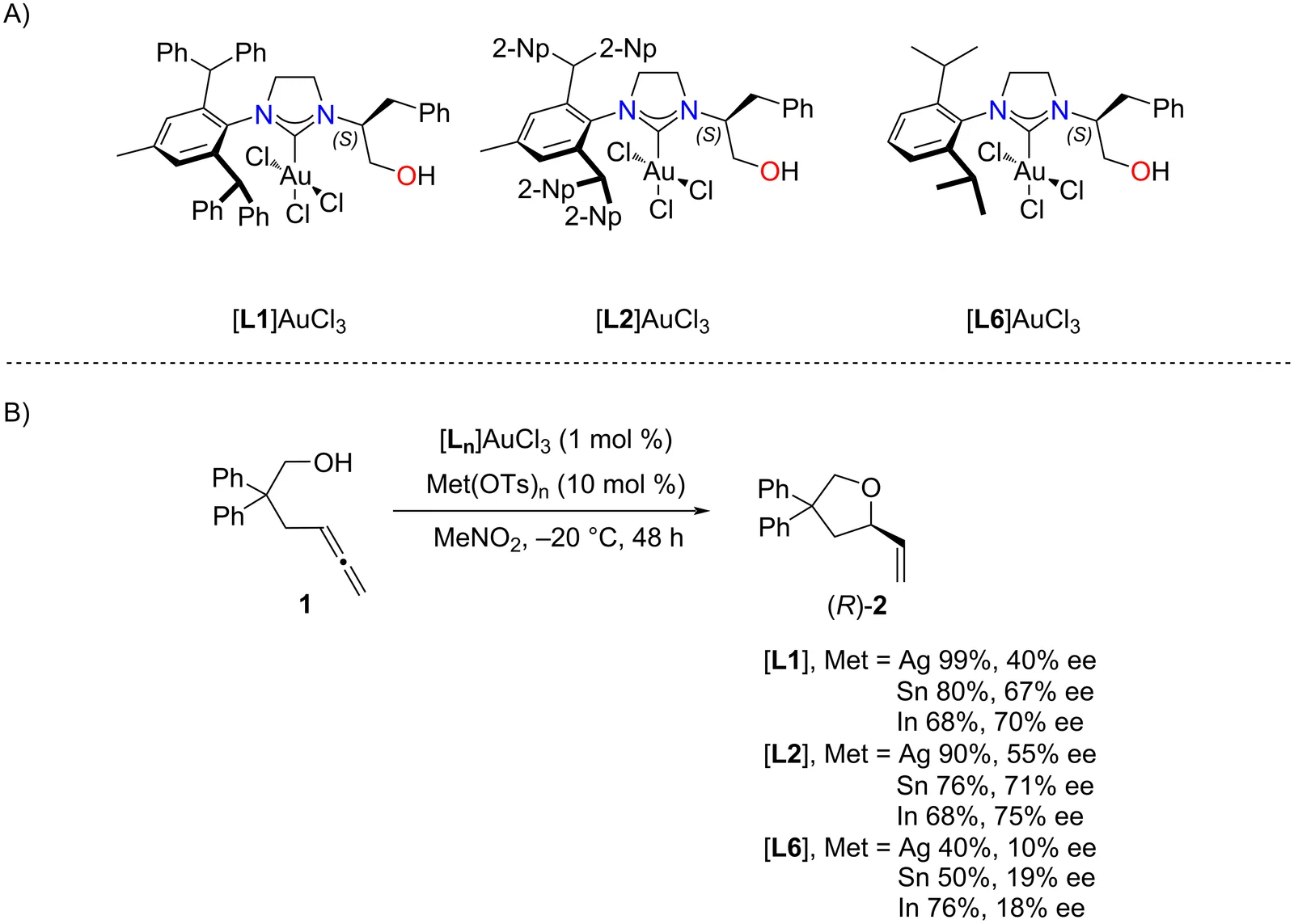
A) Structure of some chiral gold(III) complexes obtained in ref. [26]; B) cyclization of allenol **1** used as benchmark reaction to explore the catalytic performance of the same complexes with various tosylate salts.

Very recently, a second collaboration between the two groups resulted in a publication focused on chiral NHC–gold(III) complexes featuring the same ligand design employed previously [26]. The NHC-Au(III)Cl_3_ species were synthesised from the corresponding NHC-Au(I)Cl precursors by oxidation with PhICl_2_ in dichloromethane, affording the desired complexes in good to excellent yields depending on the chiral substituent attached to the NHC scaffold. The catalytic activity of the newly synthesised Au(III) complexes was subsequently investigated in the enantioselective cyclisation of allenol **1**, affording the corresponding tetrahydrofuran derivative **2**. The use of the same reaction employed for the evaluation of the chiral NHC–Au(I) complexes allowed a direct comparison of the catalytic behaviour of the two oxidation states of gold (Scheme 2).

Using similar conditions optimised in the previous study, but increasing the loading of halide scavenger, they found that the complexes featuring sterically hindered benzhydryl or naphthydryl moieties on one wingtip and a chiral phenylalaninol arm exhibited the best reactivity, affording the product in almost quantitative yields and with enantiomeric excesses of 44% (with 15 mol % AgOTs) and 55% for [**L1**] and [**L2**], respectively (Scheme 2A and B). In contrast, when the less hindered 2,6-diisopropylaniline was used as the wingtip, i.e., in [**L6**], both the yield and the enantiomeric excess of **2** decreased to 40% and 10% (with 5 mol % of AgOTs), respectively. Moreover, the formation of gold nanoparticles was observed when [**L6**] was used as the ligand, demonstrating that the role of the sterically hindered yet conformationally flexible benzhydryl or naphthydryl moiety is not limited to the creation of a three-dimensional chiral environment, but also extends to protecting the Au(III) center from reduction.

In an extended investigation aimed at identifying effective alternatives to silver salts, given the various drawbacks associated with their use [27], the authors tested nearly 30 metal tosylates in the cyclisation of **1** catalysed by [**L2**]Au(III)Cl_3_. Among the additives tested, In(OTs)_3_ and Sn(OTs)_2_ afforded tetrahydrofuran **2** with improved enantioselectivity, albeit with lower yields compared to AgOTs (Scheme 2B). Although these findings are interesting in terms of expanding the range of metal salts capable of activating gold complex precatalysts, the origin of the increased enantioselectivity remains unclear, and further mechanistic studies are required to fully elucidate its nature.

Another open question is why these Au(III) complexes display lower catalytic performance than their Au(I) counterparts, as evidenced by the yields and enantiomeric excesses obtained with [**L1**]Au(I)Cl and [**L2**]Au(I)Cl versus [**L1**]Au(III)Cl_3_ and [**L2**]Au(III)Cl_3_ in Scheme 1B and 2B, respectively. Even though some possible explanations have been proposed, further investigations are required to clarify the origin of this difference. Moreover, the presence of Au(I) species under the reaction conditions cannot be completely ruled out. These results highlight the difficulty of establishing a clear mechanistic picture for catalysis involving chiral NHC–Au(III) complexes, particularly with regard to the identity of the catalytically active species.

**Scheme 3:**
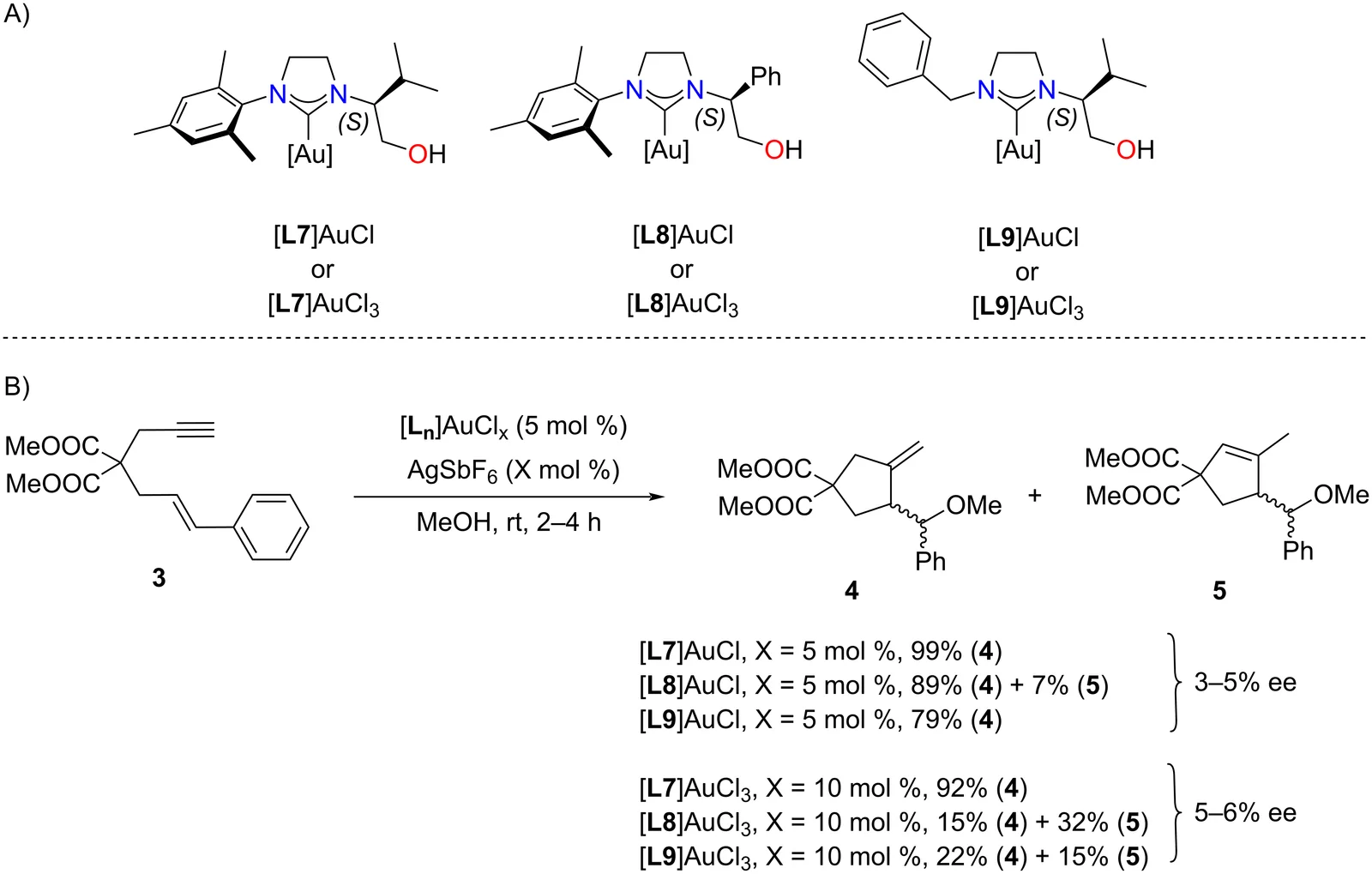
A) Structure of some chiral gold(I) and gold(III) complexes reported in ref. [28]; B) cyclization of 1,6-enyne **3** used to explore the catalytic performance of the same complexes.

This issue is further illustrated by a study reported by A. Fiksdahl and co-workers in 2021, in which a series of chiral NHC–Au(I) and NHC–Au(III) complexes were investigated in the alkoxycyclisation of 1,6-enyne **3**, using MeOH as both solvent and nucleophile (Scheme 3) [28].

For the gold(I) catalysts ([**L7**]Au(I)Cl, [**L8**]Au(I)Cl, and [**L9**]Au(I)Cl, Scheme 3A), one equivalent of AgSbF_6_ with respect to the gold complex was used (5 mol %). All complexes afforded the methoxycyclisation product **4** in excellent yields and with high selectivity, except for [**L8**]Au(I)Cl, which also gave a small amount of isomer **5**. For the gold(III) catalysts ([**L7**]Au(III)Cl_3_, [**L8**]Au(III)Cl_3_, and [**L9**]Au(III)Cl_3_, Scheme 3A), the optimal amount of silver salt was found to be 2:1 with respect to the gold complex. Lower amounts of halide scavenger resulted in reduced yields, whereas higher amounts had a negative impact on both yield and selectivity. Even under these optimised conditions, the Au(III) complexes were less efficient and selective, affording a significant amount of isomer **5** and lower yields, possibly due to the ability of Au(III) to promote a competing alkyne hydration reaction [12].

Notably, all the tested complexes, whether Au(I) or Au(III), showed very low enantioselectivity, with ee values ranging from only 3% to 6%. The lack of stereocontrol is attributed to the chiral environment provided by the ligand being too far from the reaction center, further highlighting the difficulty of achieving effective stereocontrol with these chiral NHC–gold complexes.

**Scheme 4:**
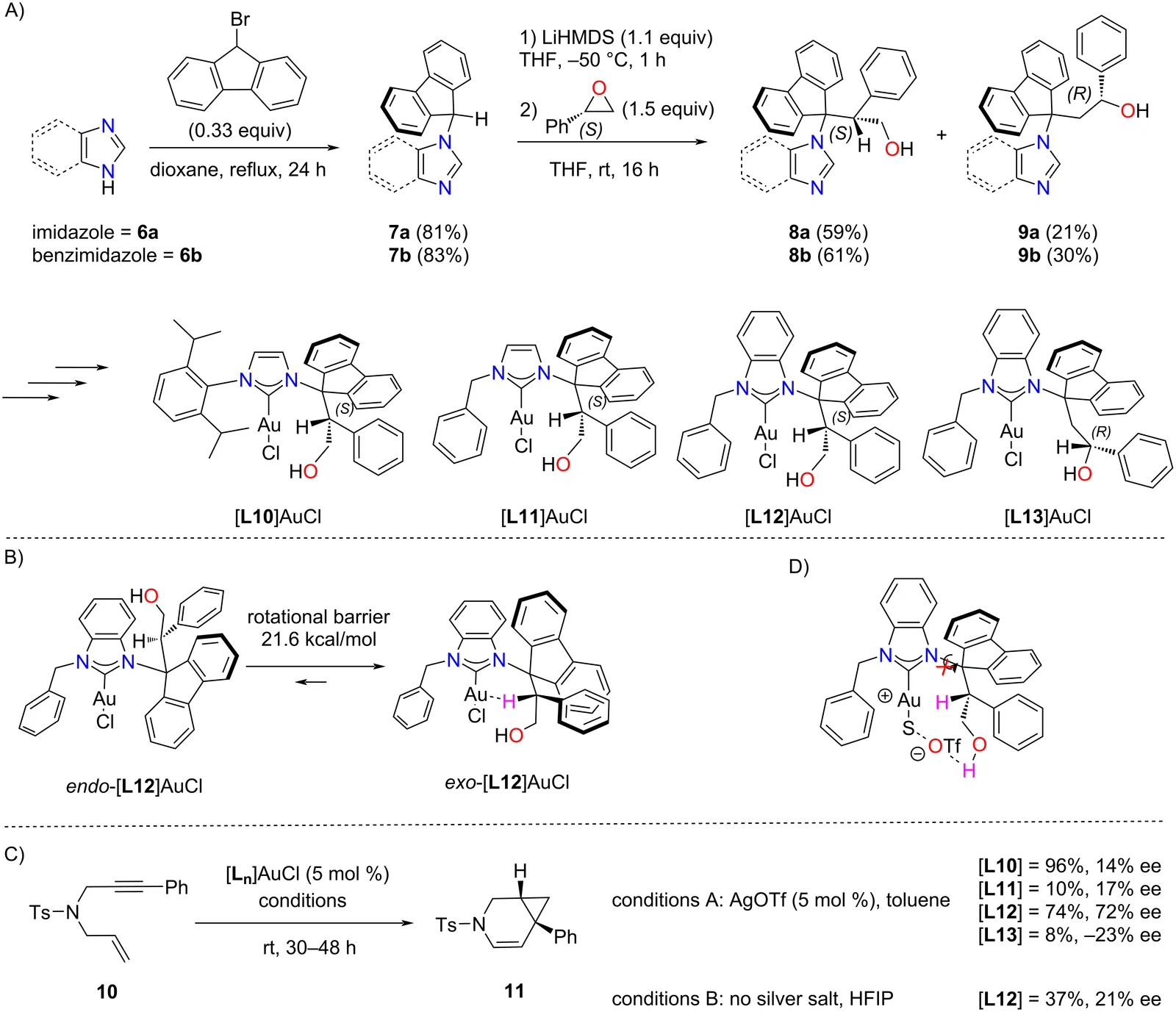
A) General synthesis of ligand precursors and structures of gold(I) complexes obtained in ref. [29]; B) representation of *endo*- and *exo*-conformation of [**L12**]Au(I)Cl; C) cyclisation of 1,6-enyne **10** used as benchmark reaction to explore the catalytic performance of the same complexes; D) postulated intermediate showing the spatial organization of the complex during catalysis.

Turning our attention back to Au(I) complexes, the use of a chiral alcohol was a strategy also followed by Blanc and co-workers, who reported the synthesis of 4 new chiral NHCs featuring a fluorenyl moiety and a benzylic alcohol arm bearing a stereogenic center (see Scheme 4A) [29].

These ligands varied in structure by the size of the core ring, being either an imidazole or a benzimidazole, and by the length of the chain bearing the stereogenic center. The corresponding gold(I) complexes were smoothly prepared and featured two rotamers: the *exo* one with the chiral chain oriented towards the gold center and the *endo* one, where the same chain points outwards. According to ^1^H NMR and confirmed by DFT calculations, due to an anagostic interaction [30] (i.e., an Au–H–C largely electrostatic interaction) between a benzylic hydrogen atom and the gold(I) center, the *exo*-conformation is favored by 5.1 kcal/mol in [**L11**]Au(I)Cl compared to the *endo* one, leading the fluorenyl group close to the NHC core. The calculated rotation barrier of the imidazole-based complex [**L11**]Au(I)Cl was 16.3 kcal/mol, while for the benzimidazole-based complex [**L12**]Au(I)Cl was 21.6 kcal/mol (Scheme 4B). This difference was attributed to the higher steric hindrance of the benzimidazole scaffold. Thus, the exchange between the two conformations is possible but slow, and only one rotamer is observed by ^1^H NMR.

The catalytic properties of the gold(I) complexes were then studied with a *N*-tosyl 1,6-enyne cyclization (compound **10**, Scheme 4C). They started the study by using 5 mol % of [**L10**]Au(I)Cl in toluene for 30 h at room temperature. The right halide scavenger was assessed screening two different silver salts: AgSbF_6_, which bears a non-coordinating anion, and AgOTf, that has a more coordinating one. Activation with silver triflate was found to be better due to stronger secondary interactions (vide infra). In these conditions, [**L10**]Au(I)Cl gave product **11** with a 96% yield and 14% ee, [**L11**]Au(I)Cl gave the same product in 10% yield and 17% ee, while [**L12**]Au(I)Cl gave 74% yield and 72% ee, confirming that the high rotational barrier imposed by the benzimidazole was beneficial. They also showed that having the stereogenic center further away from the fluorenyl, such as in [**L13**]Au(I)Cl, provided a much lower yield of 8% and an ee of −23% (the negative ee values indicate the opposite configuration of the product). Increasing the temperature up to 40 °C resulted in increased yields, but decreased ee values. Lower temperatures were not screened in this study.

These results could be explained by the formation of hydrogen bonds between the alcohol and the coordinating counteranion, locking the complex in the *exo* configuration. The substrate is thus accommodated into the chiral pocket, allowing asymmetric induction during the cyclization (see Scheme 4D). This hypothesis was confirmed by converting the alcohol into a benzylic or methyl ether. The modified catalyst yielded product **11** in a racemic mixture. Moreover, when the reaction with [**L12**]Au(I)Cl was carried out in hexafluoroisopropanol (HFIP), a solvent that is able to activate gold(I) chloride complexes in silver-free conditions [31–33], the ee of **11** dropped to 21%, a result that further supported the need of the counteranion to shift the equilibrium between the two rotamers to the most discriminating one.

Cavity-shaped ligands are increasingly valued in asymmetric catalysis for their ability to control the first coordination sphere of the metal, tuning both activity and enantioselectivity [34]. In this context, in 2013, Sollogoub and collaborators reported the synthesis of various transition metal complexes with a NHC-capped cyclodextrins (ICyD) [35]. In these systems, a NHC is installed on top of the primary rim of the cyclodextrin, pointing towards the chiral cavity of the macrocycle and therefore imposing a chiral environment around the metallic center. The cyclodextrin (CD) can thus be considered as a nanoreactor. This approach led to several publications, including gold(I) catalysis in collaboration with our group [36–40]. In all these studies, the cyclodextrin size has emerged as one of the key parameters. Indeed, three types of cyclodextrins are typically used: α, β, and γ, which differ by the number of glucose units in the ring (6, 7, and 8 units, respectively). This was shown to have a strong impact on the size of the cavity and can therefore allow for different regioselectivity compared to the use of classic NHC–metal complexes [36]. We also showed that the asymmetric CD cavity acts as a chiral nanoreactor and thus could generate a stereodiscriminative environment around the metal center important enough to induce enantioselectivity in cycloisomerization reactions of 1,6-enynes with ee values up to 80% with β-ICyD [35].

**Scheme 5:**
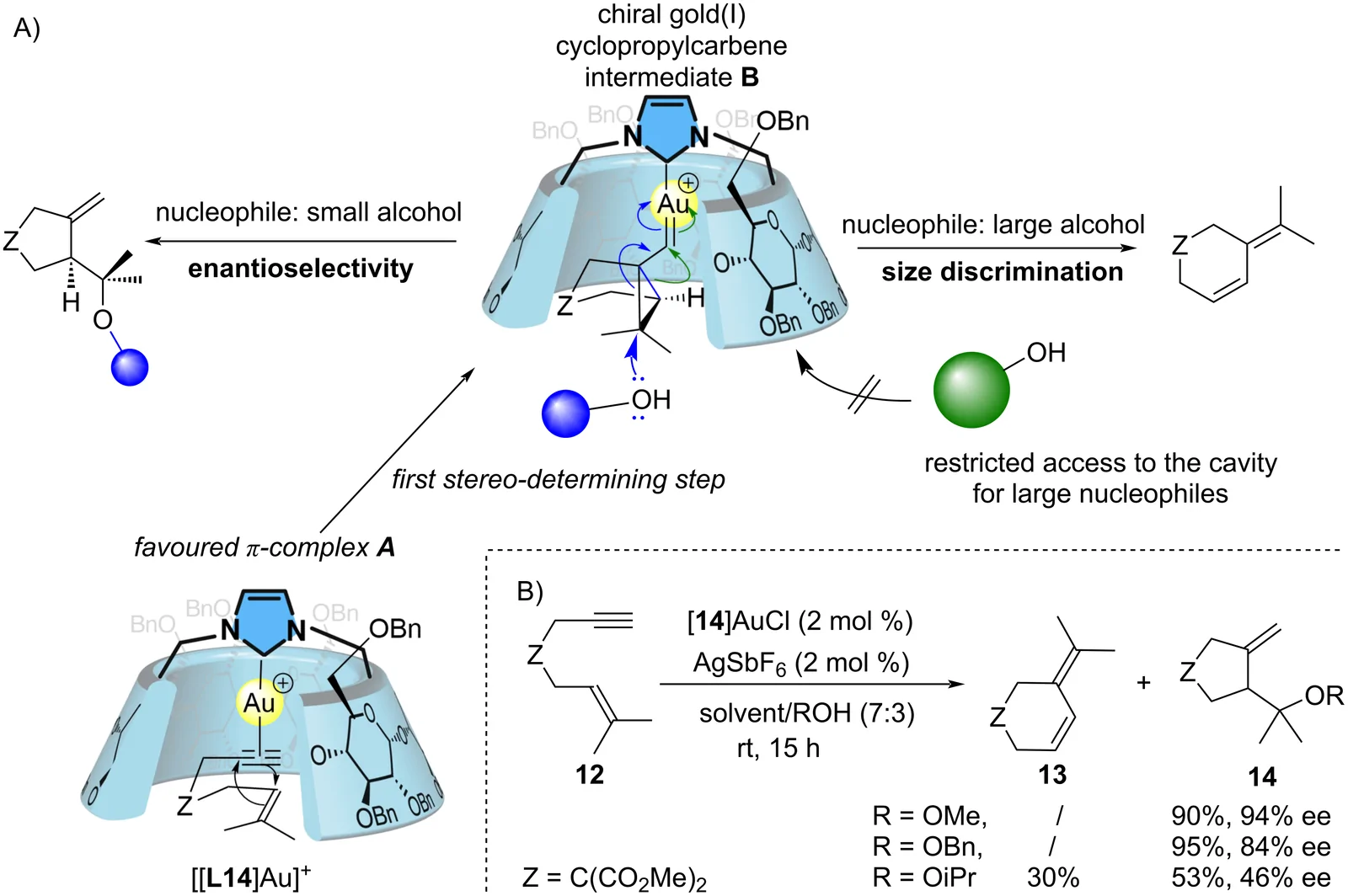
A) Proposed rationale for the observed selectivity and enantioselectivity; B) alkoxycyclization reaction results from ref. [37].

Capitalizing on these findings, we showed that the β-ICyDAuCl (i.e., [**L14**]Au(I)Cl) complex could induce a strong influence on the selectivity and enantioselectivity of an alkoxycyclization, with the outcome depending on the size of the nucleophile (Scheme 5A) [37].

Indeed, after π-coordination of the *gem*-diester enyne, the favoured π-complex **A** is formed inside the cavity. The subsequent formation of the chiral carbene intermediate **B** leads to the formation of new stereogenic center. In the presence of a large nucleophile (e.g., iPrOH), which cannot enter into the cavity, no nucleophilic attack to **B** is possible and the reaction goes toward the cyclohexadiene derivative, an outcome rarely reported in the literature (Scheme 5A) [35–36]. Regarding the alkoxycyclization reaction with smaller nucleophiles, the products were obtained in good yields and with excellent ees when MeOH and BnOH were used as nucleophiles (90% and 95% yield; 94% and 84% ee, respectively), with no formation of the cyclohexadiene derivative. Notably, the use of MeOH afforded the highest ee for product **14** ever reported for an NHC system. In contrast, a marked decrease in both selectivity and enantioselectivity was observed with iPrOH, leading to 53% of the alkoxycyclization product (46% ee) along with 30% cyclohexadiene compound formation (Scheme 5B). Of note when the reactions were conducted with N-tethered 1,6-enynes, the selectivity of the reaction was totally in favor of the alcoxycyclization pathway and the products were obtained in good yield and very high ees (84 to 88%), values never reported in the literature before.

**Scheme 6:**
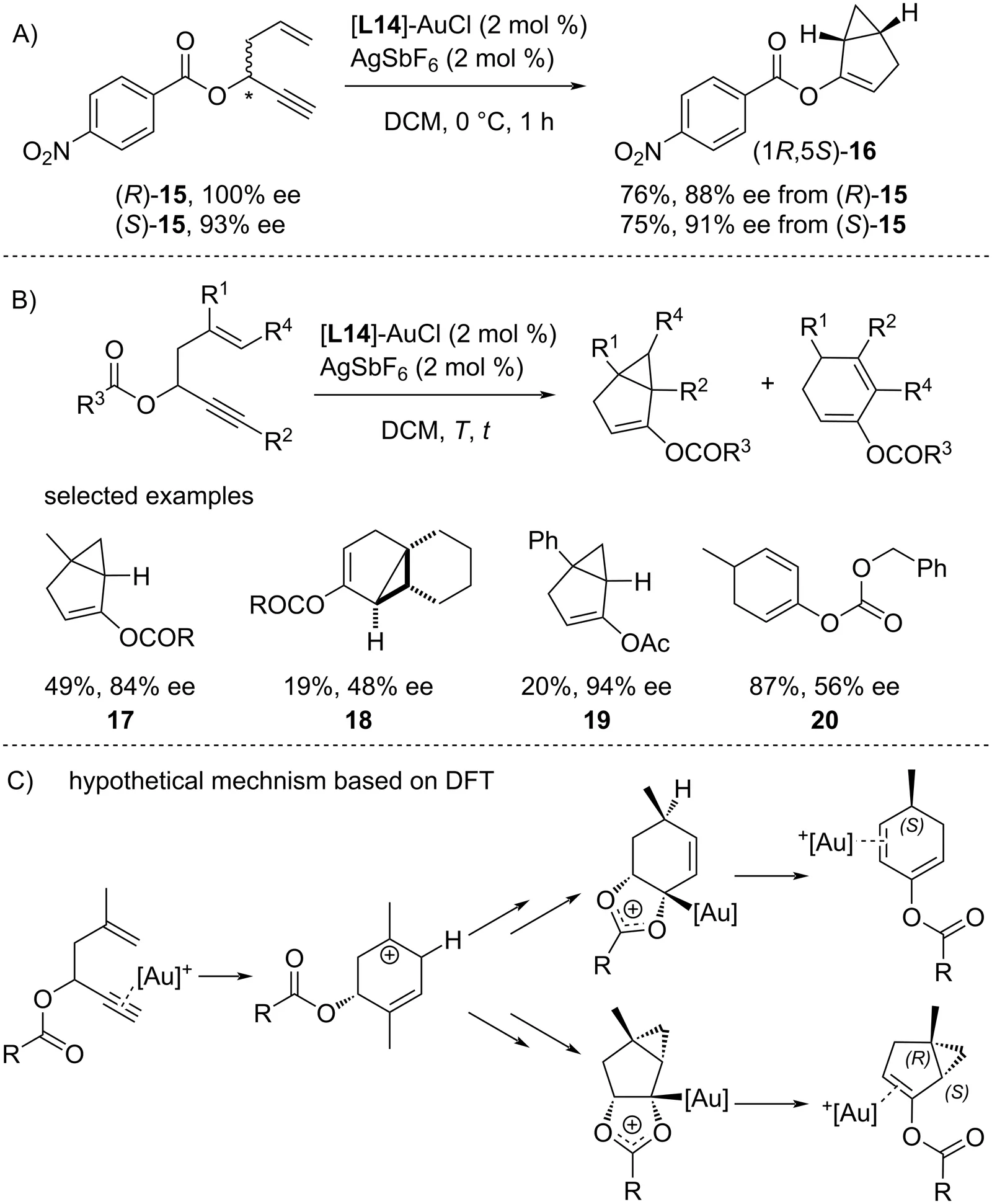
A) Cyclization of both enantiomers of 1,5-enyne **15** catalysed by **[L14]**Au(I)Cl; B) selected examples obtained by cyclization of different enynes from ref. [39]; C) DFT for the best pathway.

In 2024, using the same chiral nanoreactor (i.e., [**L14**]Au(I)Cl), our group developed a very rare enantioconvergent 1,5-enyne cycloisomerization process (Scheme 6A and 6B) [39].

In this process, the original stereochemistry of the substrate is lost because of the generation of putative carbocationic intermediate inside the cavity as suggested by DFT calculations (Scheme 6C). The stereo-outcome of the reaction would be fully controlled by the chiral environment of the cyclodextrin-based ligand [**L14**], so that both enantiomers, as well as the racemic mixture, would yield the same product with excellent enantioselectivity. A similar behaviour of this family of substrates was observed by Toste and Bohan but with lower enantioselectivity [41].

**Scheme 7:**
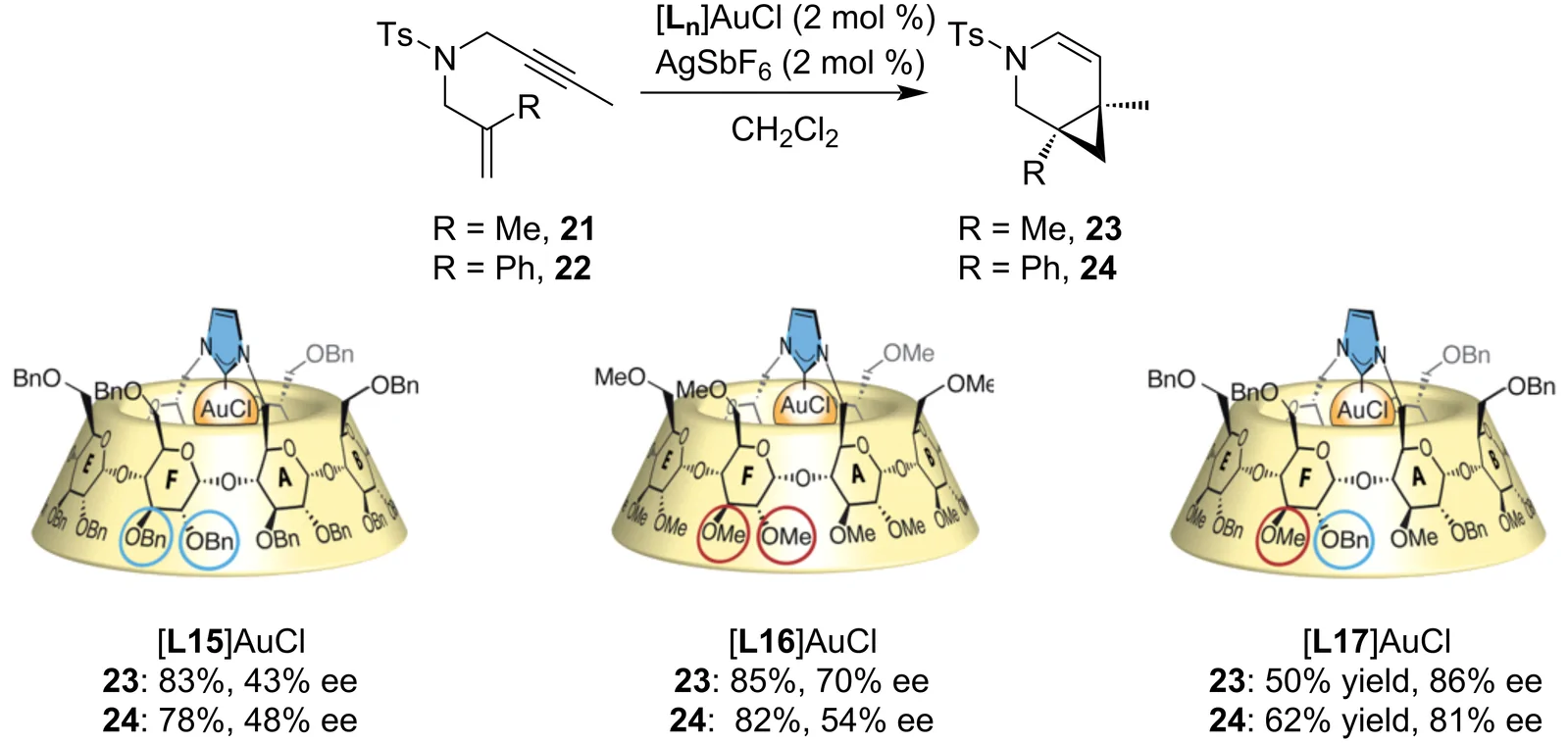
Different stereooutcomes in *N*-Ts-1,6-enyne cyclization with different cyclodextrin-based gold(I) complexes. Cyclodextrins included in Scheme 7 were reproduced from [40] (© 2025 G. Xu et al., published by Wiley-VCH GmbH, distributed under the terms of the Creative Commons Attribution-NonCommercial-NoDerivatives 4.0 International License, https://creativecommons.org/licenses/by-nc-nd/4.0/). This content is not subject to CC BY 4.0".

As we demonstrated in a subsequent work, modification of the secondary rim of the cyclodextrin could affect the enantioselectivity but also the reaction rate of the cycloisomerization of two slightly different *N*-tosyl-1,6-enynes (Scheme 7) [40].

Indeed, with smaller OMe groups in [**L16**]Au(I)Cl, the reaction had a faster rate due to an easier deformation of the cavity, allowing the substrate to enter more efficiently. The smaller methoxy groups also increased the enantiomeric ratios in both reactions. Surprisingly, even better results were obtained when the two protecting groups were mixed (OMe on O2 and OBn on O3) in [**L17**]Au(I)Cl, yielding products **23** and **24** in lower yields but higher ees (see Scheme 7). This is likely due to a mixed effect of the substituent allowing an easier deformation of the cavity while keeping a sterically constrained environment.

### ImPy–gold(I) complexes

Imidazo[1,5-*a*]pyridin-3-ylidenes, commonly known as ImPy carbenes, are a class of NHC ligands independently reported in 2005 by the groups of Lassaletta and Glorius [42–43]. These highly modular ligands can be easily and independently functionalized at the N(2) and C(5) positions with sterically demanding groups or chiral appendages. Unlike “classical” NHCs, such as 1,3-disubstituted imidazolylidenes, where the N-wingtips generally exert limited influence on the metal coordination sphere, the rigid bicyclic structure of the ImPy scaffold places the substituent at C(5) in close proximity to the metal center [44]. As a result, remote substituents can exert a strong influence on the chiral environment around the metal and, in some cases, participate in secondary interactions with substrates or reaction intermediates, ultimately affecting catalytic activity and enantioselectivity. In this respect, ImPy ligands are somewhat reminiscent of Buchwald-type dialkylbiarylphosphines, where remote steric and electronic effects play a crucial role in catalyst performance [45–46]. Moreover, similarly to “classical” NHCs, ImPy carbenes are strong σ-donor ligands, efficiently stabilizing electron-deficient metal centers and often providing catalytic profiles distinct from those of phosphine-based systems [47].

Since their first report, several chiral ImPy-based gold complexes have been developed by different research groups, taking advantage of their versatility, robustness, and unique steric properties [48–51]. In line with the scope of this review, the following section summarizes the most significant advances in asymmetric gold catalysis based on chiral ImPy ligands, starting from the work reported by Zhang and co-workers in 2019 [24].

**Scheme 8:**
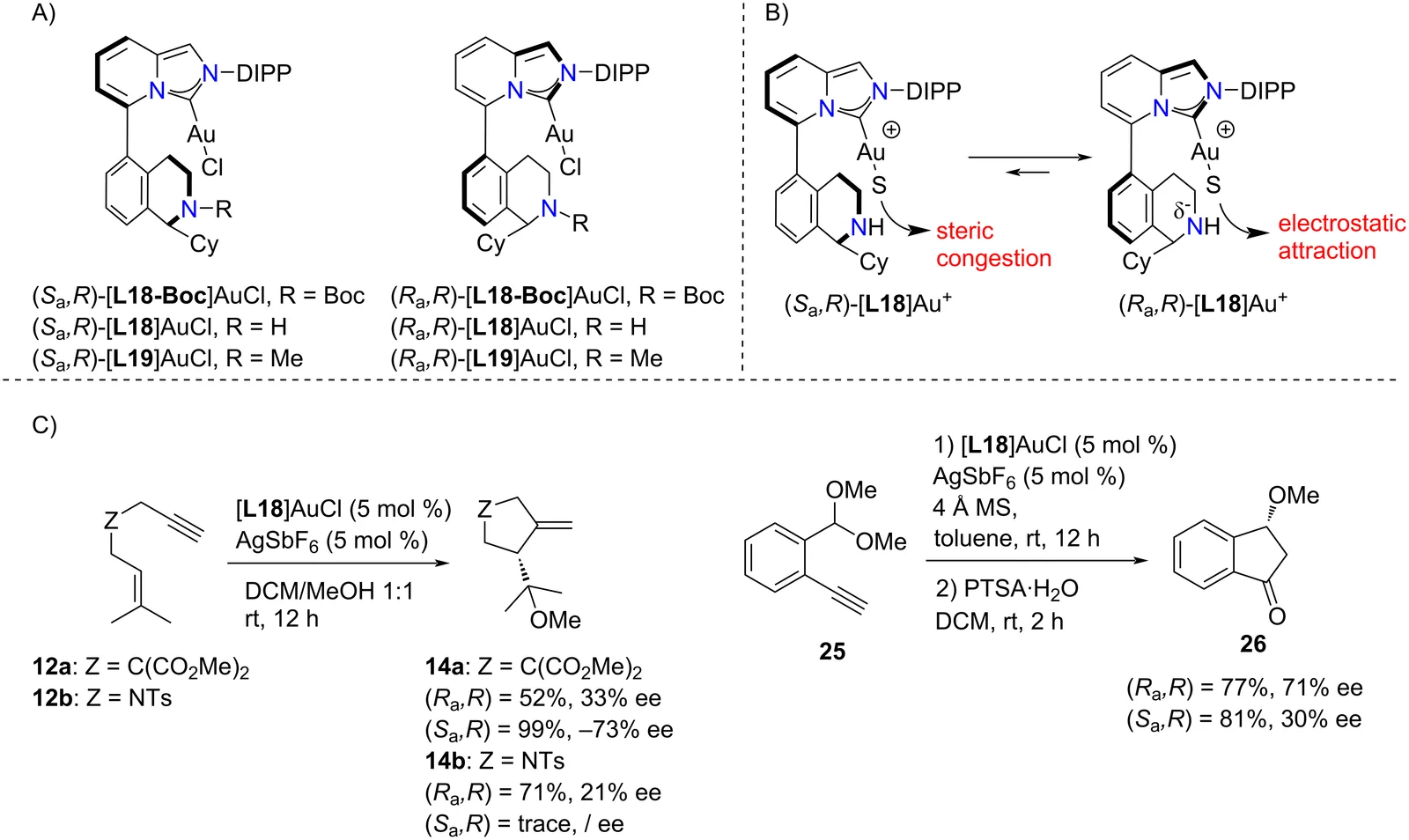
A) Structures of complexes obtained in ref. [24] (DIPP = 2,6-diisopropylphenyl group); B) fluxional equilibrium in cationic [**L18**] gold complex (S = solvent or substrate); C) alkoxycyclization of 1,6-enynes and carboxyalkylation of alkyne **25**, two benchmark reactions explored in the study.

Based on their experience in the development of remotely functionalized biaryl-2-ylphosphines [52–53], Zhang and co-workers developed a family of gold complexes supported by ImPy ligands functionalized at C(5) with a chiral tetrahydroisoquinoline moiety (Scheme 8). All the complexes investigated in this study originated from a common NHC precursor that, after metalation, produced the gold complex [**L18-Boc**]Au(I)Cl as a mixture of diastereomeric atropoisomers, which could be readily separated by silica gel flash column chromatography. The Boc protecting group was then removed to obtain [(*S*_a_*,R*)-**L18**] and [(*R*_a_*,R*)-**L18**]Au(I)Cl quantitatively. Condensation of paraformaldehyde allowed the isolation of [(*S*_a_,*R*)-**L19**] and [(*R*_a_,*R*)-**L19**]Au(I)Cl in very high yields (Scheme 8A). Since the restricted rotation of the biaryl axis is due to the presence of Au(I)Cl moiety, they investigated whether chloride removal could favor rotation and thus lead to a loss of axial enantiomeric excess. [(*S*_a_,*R*)-**L18**]Au(I)Cl was therefore cationized with NaBArF, heated, and then quenched with TBACl to restore the original neutral complex. A small amount of the (*R*_a_,*R*) atropoisomer was already visible at room temperature, with a further increase to a 1.00/0.04 ratio after 24 hours at 80 °C (equilibrium point). The same ratio was reached starting from enantiopure [(*R*_a_*,R*)-**L18**]Au(I)Cl, confirming the fluxional nature of the ligand. The (*R*_a_,*R*) configuration appears to be thermodynamically more stable due to a favorable electrostatic attraction between the cationic gold center and the partially negative nitrogen atom. This interaction is not present in the (*S*_a_,*R*) atropoisomer, where the cyclohexyl group points towards the gold center, making it thermodynamically less stable (Scheme 8B). Axial epimerization was also detected in heated solutions of enantiopure [**L18**]Au(I)Cl, with rates similar to those observed in the cationic gold experiments. However, the atropoisomers were stable in the solid state when stored at 0 °C.

The complexes were then tested in a series of asymmetric gold-catalyzed reactions. [**L18**]-based complexes performed better than their methylated counterparts [**L19**], providing superior yields and ee values. Despite the fluxional nature, the [(*S*_a_,*R*)-**L18**]Au(I)Cl precomplex gave generally slightly better results than its atropoisomer, except in the alkoxycyclization of N-tethered 1,6-enynes and in the carboalkoxylation of alkynes (Scheme 8C). The authors concluded that both attractive interactions in (*R*_a_*,R*) and steric repulsions in (*S*_a_*,R*) can provide enantioinduction, depending on the reaction under consideration.

**Scheme 9:**
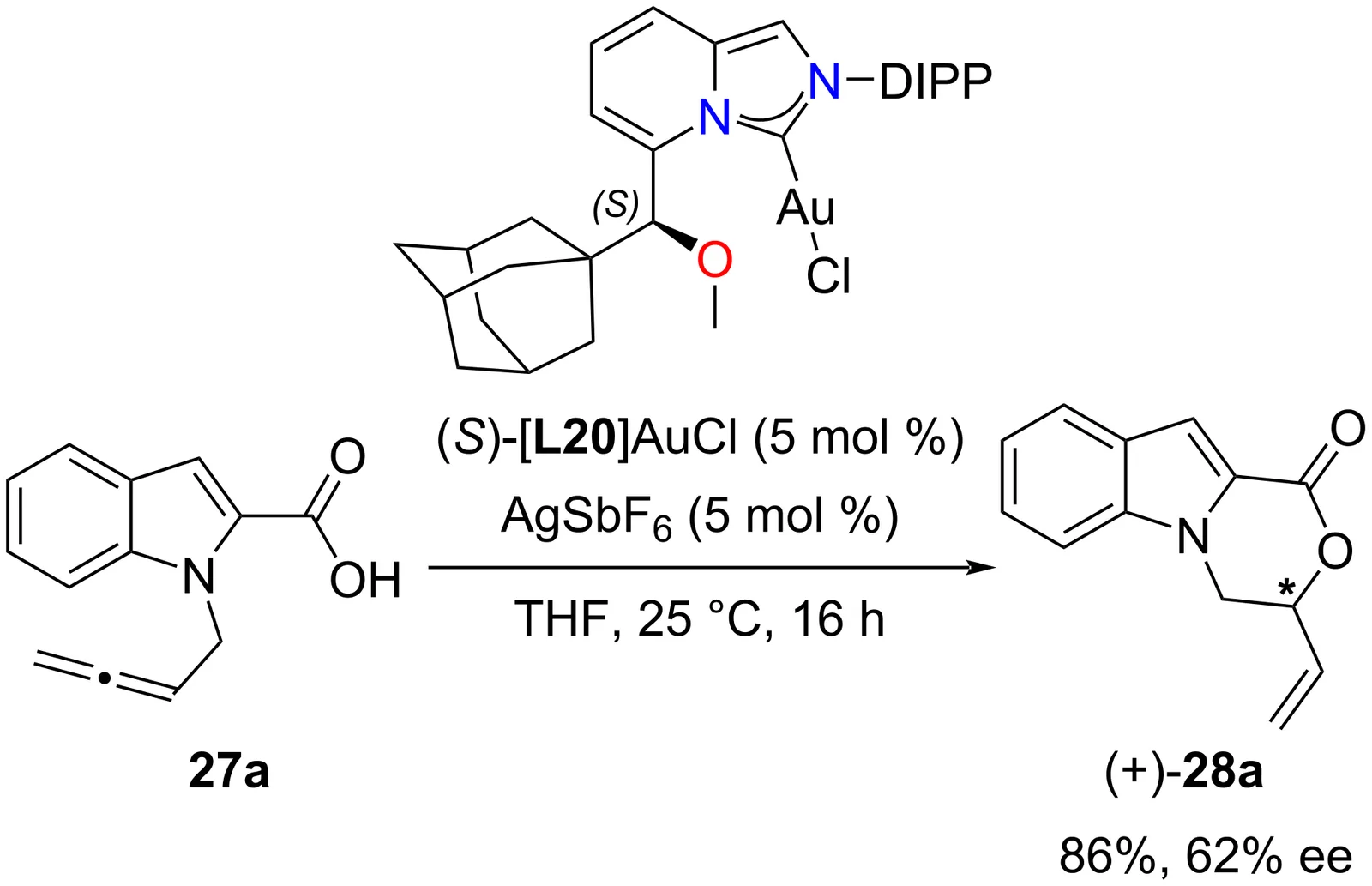
Enantioselective synthesis of 3,4-dihydro-1*H*-[1,4]oxazino[4,3-*a*]indol-1-one **28a** by precatalyst [(*S*)-**L20**]Au(I)Cl as reported in ref. [54].

In 2022, Monari, Ollevier, Bandini, and co-workers reported the gold(I)-catalysed intramolecular hydrocarboxylation of allenes, a novel reaction that enabled the synthesis of tricyclic N(1)-C(2)-fused oxazino-indolones (Scheme 9) [54].

For this particular transformation, the catalyst of choice was a family of ImPy-based gold(I) complexes with various substituents at C(5). Among six different achiral complexes, the one bearing an electron-poor 3,5-(CF_3_)_2_-aryl ring exhibited the highest efficiency, leading to product **28a** in 90% yield. Interestingly, common NHC- and phosphine-based gold complexes such as IPrAuCl and Ph_3_PAuCl proved significantly less active, affording yields of 69% and 42%, respectively. The exceptional performance of the ImPy complex was proposed to arise from destabilization of the cationic organogold intermediates by the electron-poor arene located close to the metal center [55]. The ImPy core was then modified at C(5) with enantiomerically pure secondary alcohols in order to develop the asymmetric variant of the same reaction. Four different complexes were thus prepared, featuring *t-*Bu and adamantyl substituents at the carbinol site together with variously substituted oxygenated moieties (i.e., -OH, -OMe, and -OAc groups). Very high yields of the product were obtained in all cases after a 16 h reaction using AgSbF_6_ as chloride scavenger in THF at 25 °C (80–90%). In terms of enantiomeric excess, the catalyst bearing the free alcohol proved to be the least effective, affording (−)-**28a** in 34% ee, whereas the adamantyl-substituted analogue bearing an -OMe group (i.e., [(S)-**L20**]Au(I)Cl) gave the best result, yielding (+)-**28a** in 62% ee.

**Scheme 10:**
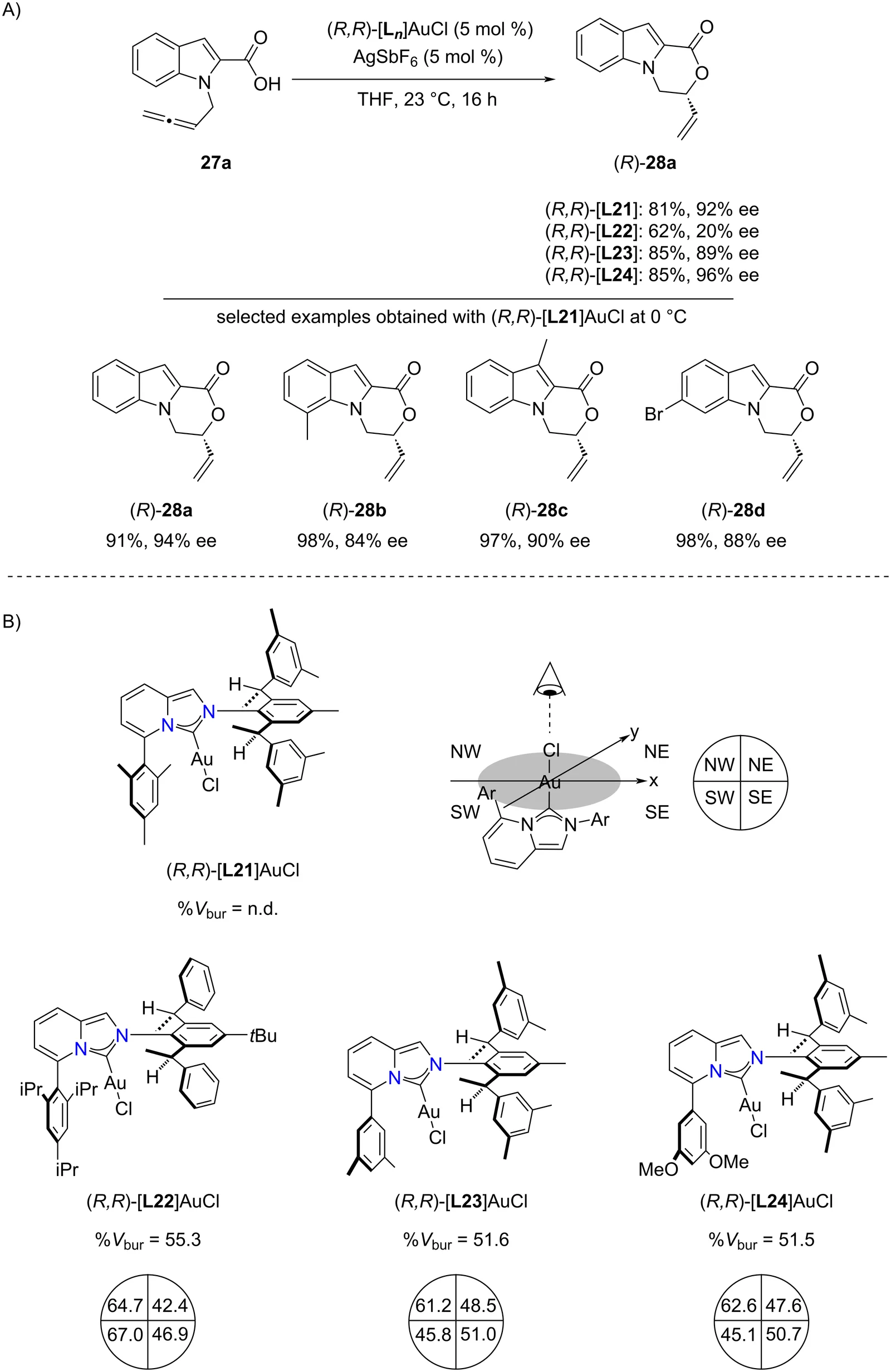
A) Enantioselective synthesis of 3,4-dihydro-1*H*-[1,4]oxazino[4,3-*a*]indol-1-one **28a** and selected examples of variously substituted products obtained by different precatalysts in ref. [56]; B) structure and analysis of steric maps of four gold(I) complexes developed in the same study.

The same reaction was explored a few years later by the group of Mazet in a study focused on the development of gold(I) complexes based on ImPy ligands featuring chiral aniline moieties at N(2) (Scheme 10) [56]. Using 2,6-di(1-arylethyl)aniline derivatives [57], they synthesized ten different ligands and their corresponding gold(I) complexes in four steps, with overall yields ranging from 23% to 62%. Screening of the complexes in the above-mentioned enantioselective intramolecular hydrocarboxylation revealed that the best balance between reactivity and enantioselectivity was achieved with a mesityl unit at C(5) (i.e., (*R,R*)-[**L21**]Au(I)Cl), affording an 81% yield and a 92% ee (Scheme 10A). Further improvement was observed when the reaction was conducted at 0 °C, providing the product in 91% yield and 94% ee. Interestingly, contrary to the observations reported by Bandini and co-workers, the introduction of a 3,5-(CF_3_)_2_-aryl ring at C(5) reduced the reactivity while maintaining a comparable enantiomeric excess (53% yield and 86% ee at rt). Analysis of steric maps for buried volume (%V_bur_) [58–62], obtained from single crystal X-ray diffraction (SCXRD) analysis of three complexes (i.e*.,* [**L21**]Au(I)Cl, [**L22**]Au(I)Cl, [**L23**]Au(I)Cl), suggested that the high level of enantioinduction originates, at least in part, from a pronounced difference in buried volume among the four quadrants (see Scheme 10B). In contrast, the complex displaying only moderate enantioinduction showed little difference between the NW and SW quadrants (both crowded), as well as between the NE and SE quadrants (both open). The scope of the asymmetric reaction was finally evaluated under the optimized conditions using precatalyst [(*R,R*)-**L21**]Au(I)Cl. The Scheme 10A highlights representative examples of oxazino-indolones synthesized in this study. Remarkably, methyl groups at sterically demanding positions (i.e., C(7) and C(3) in (*R*)-**28b** and (*R*)-**28c**, respectively) did not affect the catalytic performance, nor did halides at the C(4) or C(5) positions. The absolute configuration of (*R*)-**28d** was determined by SCXRD analysis, while those of the remaining products were assigned by analogy.

**Scheme 11:**
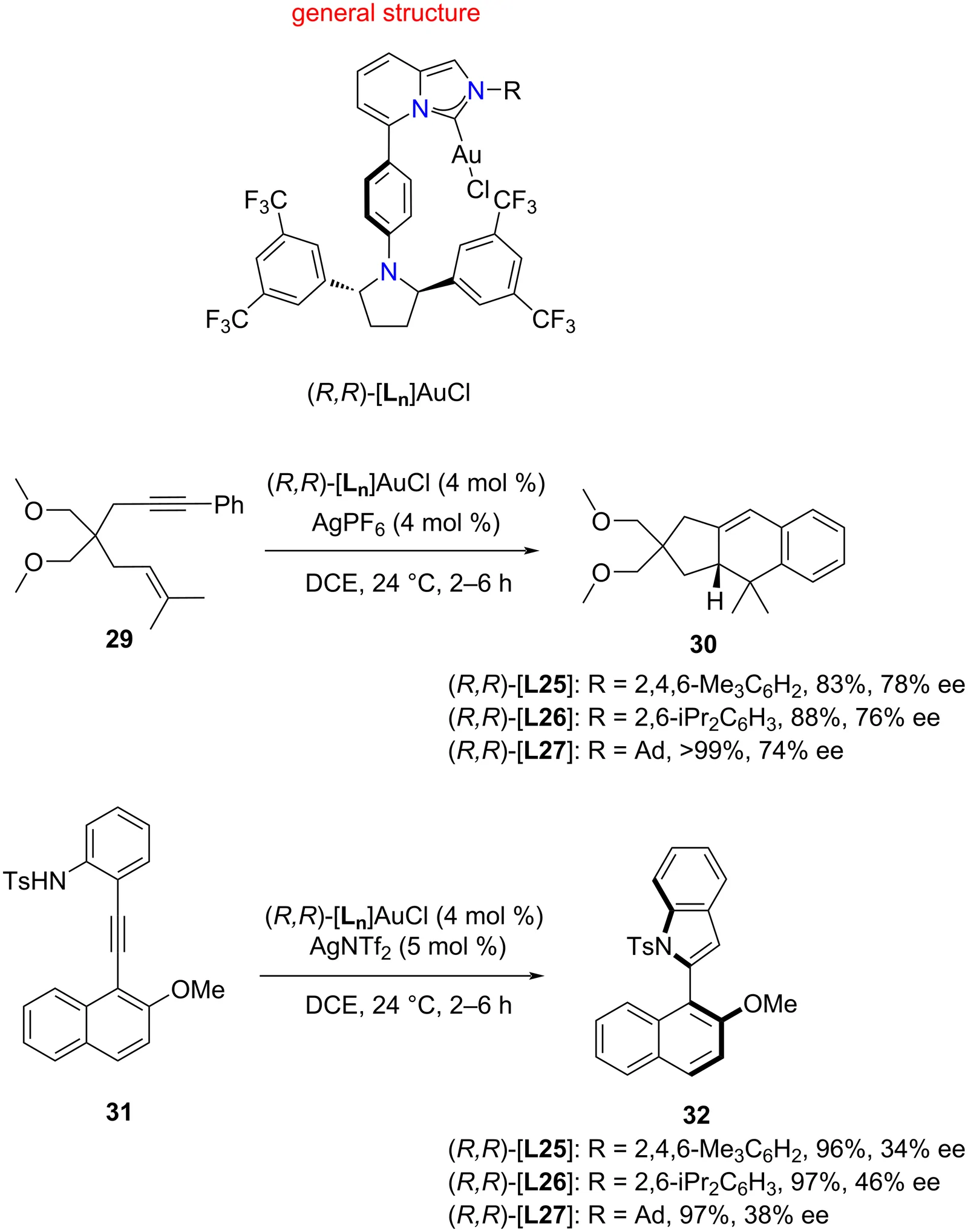
General structure of ImPy precatalysts obtained in ref. [6] and their outcomes in asymmetric [4 + 2] cycloaddition of enyne **29** and atroposelective cyclisation of sulphonamidyl diarylacetylenes **31**.

In a study focused on the development of a new generation of chiral gold(I) complexes based on modifications of the JohnPhos scaffold, the groups of Besora, Escofet, and Echavarren reported the synthesis of three ImPy-based complexes featuring a remote *C*_2_-symmetric chiral 2,5-diarylpyrrolidine unit (Scheme 11) [6]. The ligands differed only in the N(2) substituent, which consisted either of a sterically hindered arene [(*R,R*)-**L25**]Au(I)Cl and [(*R,R*)-**L26**]Au(I)Cl, or a bulky adamantyl group [(*R,R*)-**L27**]Au(I)Cl. These complexes were tested in the benchmark [4 + 2] cycloaddition of enyne **29**, as well as in the atroposelective cyclisation of sulphonamidyl diarylacetylenes **31** to form 2-arylindole **32**. In both cases, the ImPy-based ligands provided yields comparable to those obtained with several phosphine ligands, although the enantiomeric ratios were not particularly competitive.

During the computational analysis of buried volumes, the authors have shown that an elongated shape provided a more accurate description of the gold(I) catalysts under investigation. They therefore developed the NEST tool, an open-source web program designed to evaluate the steric effects of bulky ligands in metal complexes. NEST defines a non-spherical volume around the metal center, referred to as a capsule-shaped nest, and calculates the percent volume occupied by the atoms (i.e*.*, the NEST occupied volume). This volume can also be divided into quadrants to determine the occupied volume in each one. Finally, the authors developed a mathematical model to reproduce and predict enantiomeric ratios based on structural analyses performed using NEST. Although the derived equations were able to predict the experimental results for phosphine-based complexes with reasonable agreement, the same level of accuracy could not be achieved for ImPy-based ligands.

**Scheme 12:**
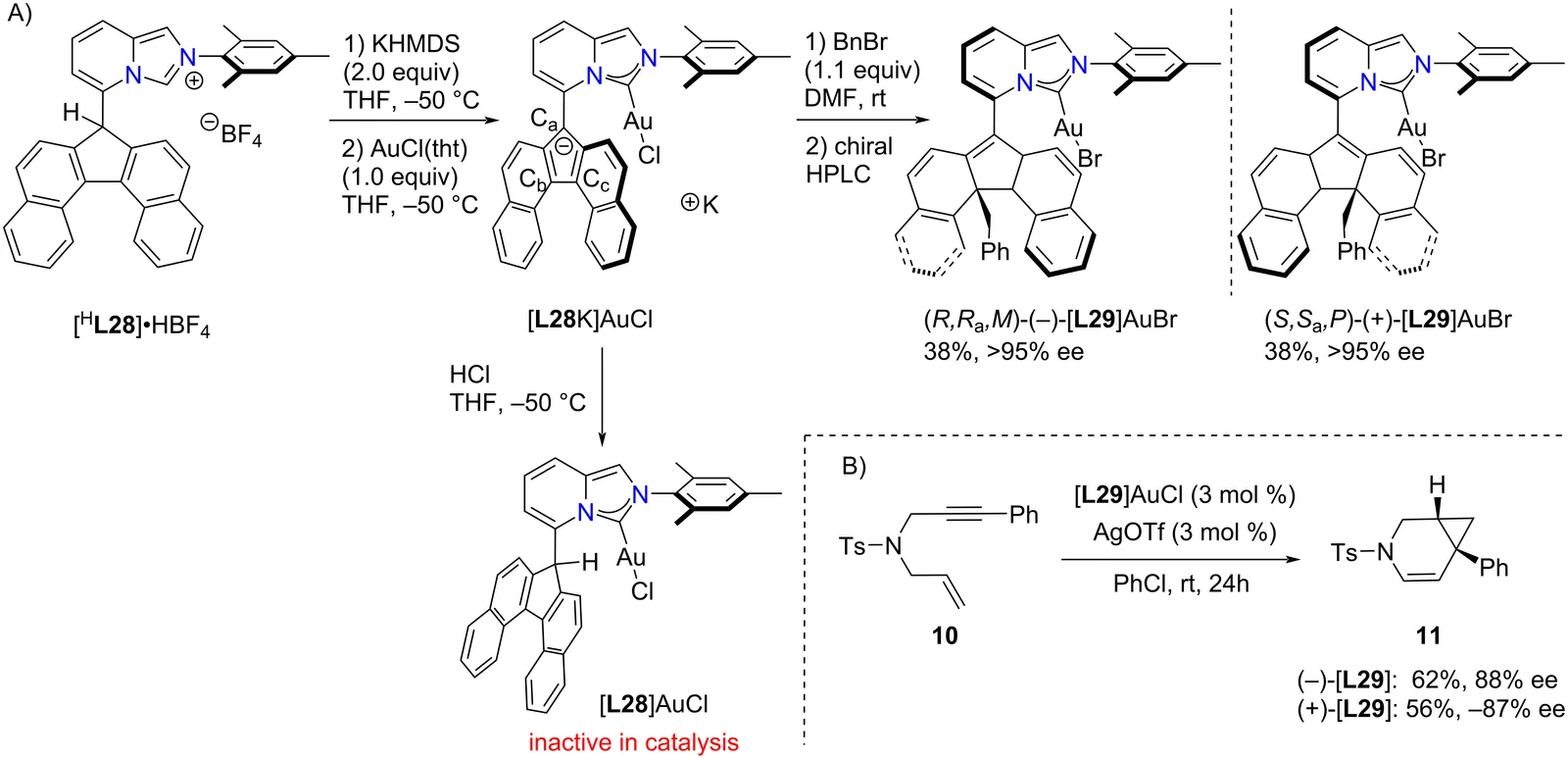
A) Synthesis of [**L28**]Au(I)Cl and (±)[**L29**]Au(I)Br; B) outcome in asymmetric cycloisomerization of N-tethered 1,6-enyne **10**. From ref. [63].

In 2022, the groups of Autschbach, Crassous, Bastin, and César described the synthesis of an ImPy-based gold(I) complex combining central, axial, and helical chirality (Scheme 12) [63].

The study began with the grafting of a fluorenyl-derived pentahelicenic moiety at C(5) of a classical imidazo[1,5-*a*]pyridinium scaffold bearing a mesityl substituent at N(2). The resulting salt [**^H^L28**]·HBF_4_ was then deprotonated with two equivalents of potassium bis(trimethylsilyl)amide (KHMDS), and the carbene trapped with tetrahydrothiophene gold(I) chloride complex (AuCl(tht)). Subsequent re-protonation of the anionic gold(I) intermediate afforded the neutral complex [**L28**]Au(I)Cl in excellent yield (Scheme 12A). SCXRD analysis revealed that the helicene points away from the gold center, while the fluorenyl-type C–H bond is positioned close to the metal at a distance of only 2.634 Å. This geometry suggested the presence of a weak “contra-electrostatic H bond”. However, this complex proved inactive as a precatalyst in the classical gold-catalyzed cycloisomerization of N-tethered 1,6-enyne **10**, undergoing complete decomposition, probably because of the close proximity of the acidic fluorenyl proton. The authors, therefore, attempted benzylation at that position (i.e., C_a_ in [**L28K**]Au(I)Cl; see Scheme 12A). Surprisingly, benzylation occurred instead at C_b_ or C_c_ positions. This unexpected reactivity generated a complex with three distinct stereogenic elements simultaneously: central chirality at the sp^3^ carbon, axial chirality around the axis connecting the ImPy core to the helicene, and helical chirality arising from the helicene framework itself ((±)-[**L29**]Au(I)Br). The two enantiomers were separated by chiral preparative HPLC, and their absolute configurations were confirmed by SCXRD analysis. The enantiopure complexes were again evaluated as precatalysts in the asymmetric cycloisomerization of N-tethered 1,6-enyne **10**. After chloride abstraction with AgOTf in chlorobenzene, both complexes provided moderate yields and excellent ee values, giving opposite stereoisomers depending on the chirality of the gold complex (Scheme 12B). The reaction tolerated both electron-withdrawing and electron-donating *para*-substituents on the aryl groups of the 1,6-enyne, as well as bulkier 1-naphthyl substituents.

**Scheme 13:**
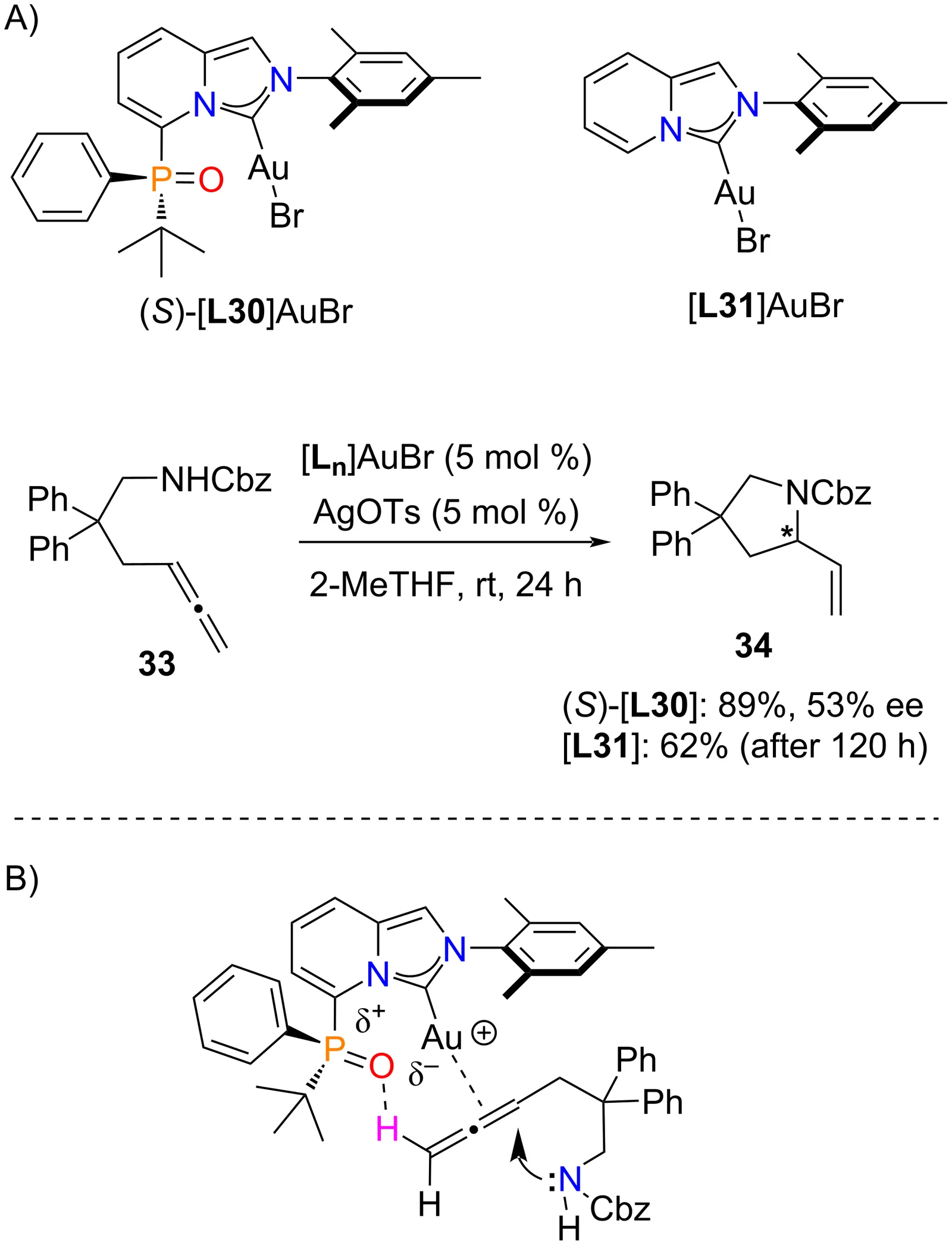
A) Structure of precatalysts [(*S*)-**L30**]Au(I)Br and [**L31**]Au(I)Br and their outcomes in asymmetric intramolecular hydroamination of a *N*-allenyl carbamate **33**; B) proposed reaction intermediate showing the secondary interactions between the complex and the substrate. From ref. [64].

In 2025, Tsantrizos et al. reported the synthesis of an ImPy Au(I)Br precatalyst with a *P*-chiral phosphine oxide at C(5) (Scheme 13) [64].

Similar to the idea of bifunctional ligand developed by Zhang, the authors wanted to exploit the P-stereogenic phosphine oxide in [(*S*)-**L30**]Au(I)Br to generate a chiral environment in close proximity to gold and also to act as a Lewis base and introduce beneficial weak electrostatic interactions in asymmetric catalysis. To prove it, a second precatalyst missing the chiral phosphine oxide was also prepared ([**L31**]Au(I)Br, Scheme 13A). The intramolecular hydroamination of the *N*-allenyl carbamate **33** was chosen as benchmark reaction. Using AgOTs as halide scavenger, the chiral catalyst converted the substrate quantitatively after 24 h in DCM, 2-MeTHF and toluene. The best ee was 53% in 2-MeTHF, although it was close to the one obtained in toluene and DCM (52% and 40%, respectively). The catalyst missing the phosphine oxide performed considerably slower, with a low conversion of 62% after 120 hours in 2-MeTHF. The mechanism of the reaction was already elucidated by Toste and co-workers, who identified the formation of the π-Au(I) complex as the rate-determining step (Scheme 13B) [65]. DFT calculations provided important insights about the reaction mechanism with the bifunctional ligand. The efficiency of this catalyst relies on an interaction between the P=O moiety and the terminal protons on the allene that strengthen the binding of the latter to gold. Owing to this hydrogen-bonding interaction, the rate determining step was calculated to be 18.9 kJ/mol more favorable with [**L30**] than with [**L31**], leading to a faster reaction as confirmed by experimental data.

**Scheme 14:**
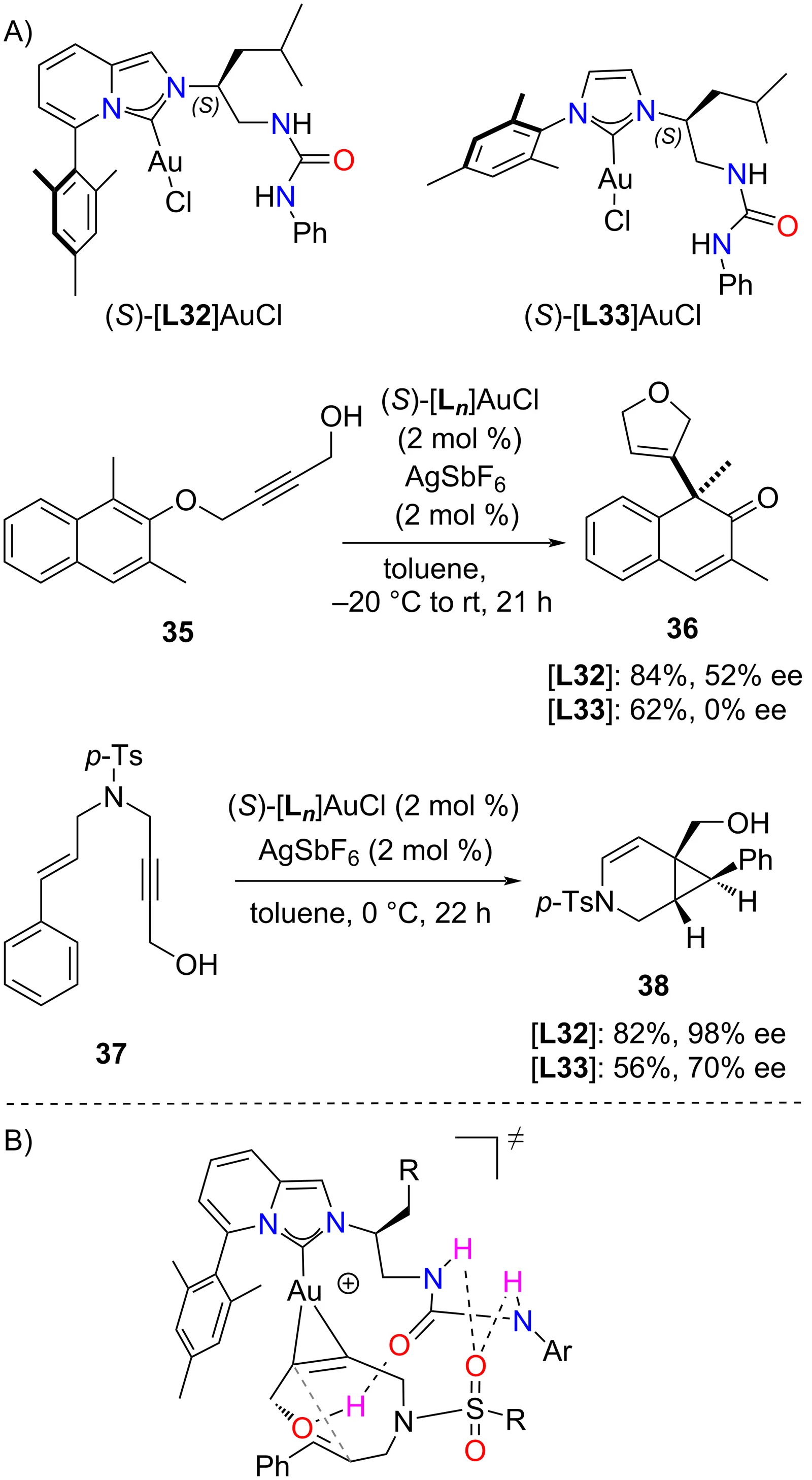
A) Structure of precatalysts (*S*)-[**L32**]Au(I)Cl, (*S*)-[**L33**]Au(I)Cl, and their outcomes in asymmetric dearomatization of 2-naphthol **35** and cyclisation of 1,6-enynol **37** from ref. [66]; B) secondary interactions in the transition state of *Si*-face attack.

In collaboration with the group of Helaja, our group recently reported the development of a new family of ImPy-based gold(I) complexes bearing a chiral arm at N(2), derived from the α-amino alcohols ʟ-leucinol and ʟ-phenylalaninol (Scheme 14) [66].

A mesityl ring was retained at C(5), while the chiral arm was further functionalized with a urea moiety bearing different aryl substituents at the terminal nitrogen atom. In this way, five gold complexes were synthesized over seven steps, with overall yields ranging from 10% to 25%. Initial screening of selected precatalysts in a benchmark cyclisation of allenols showed high yields but modest enantioselectivities. The attention was therefore shifted to the dearomatization of 2-naphthol derivative **35**, a transformation recently reported by Bandini et al. [67]. Precatalyst (*S*)-[**L32**]Au(I)Cl delivered the best results in the presence of AgSbF_6_ in toluene at −20 °C. Interestingly, a related precatalyst featuring the same chiral arm attached to the nitrogen atom of a “classical” imidazolyl NHC ligand afforded comparable yields but proved completely ineffective in terms of enantioinduction (see (*S*)-[**L33**]Au(I)Cl in Scheme 14A). (*S*)-[**L32**]Au(I)Cl also delivered the best results in the 6-*endo*-*dig* cyclisation of 1,6-enynol **37**, affording product **38** in 89% yield and 94% ee. Running the reaction at 0 °C further improved the ee to 98%. Lower enantioselectivities were observed in polar solvents or when using AgOTs, probably because of disruption of secondary interactions between the substrate and the chiral arm of the catalyst. This hypothesis was supported by DFT calculations, which revealed H-bonding interactions between the hydroxy group of the substrate and the carbonyl of the urea during the first transition state associated with C–C bond formation (Scheme 14B). These interactions lowered the energy barrier for *Si*-face attack relative to *Re*-face attack. This result is fully consistent with the formation of (1*S,*6*R,*7*R*)-**38** as essentially the sole enantiomer, as confirmed by SCXRD analysis. This work emphasizes how the presence of non-covalent interactions can assist enantioinduction in asymmetric gold(I) catalyzed cyclization.

### Miscellaneous NHC–gold(I) complexes

In this part, NHC that do not fall into the “classical” NHC or ImPy categories will be discussed. Indeed, many other types of NHC can be found in the literature [68–73], but only few examples as ligands for gold complexes [74–79]. Still, some research has been recently done with non-traditional NHCs and will be presented in this chapter.

**Scheme 15:**
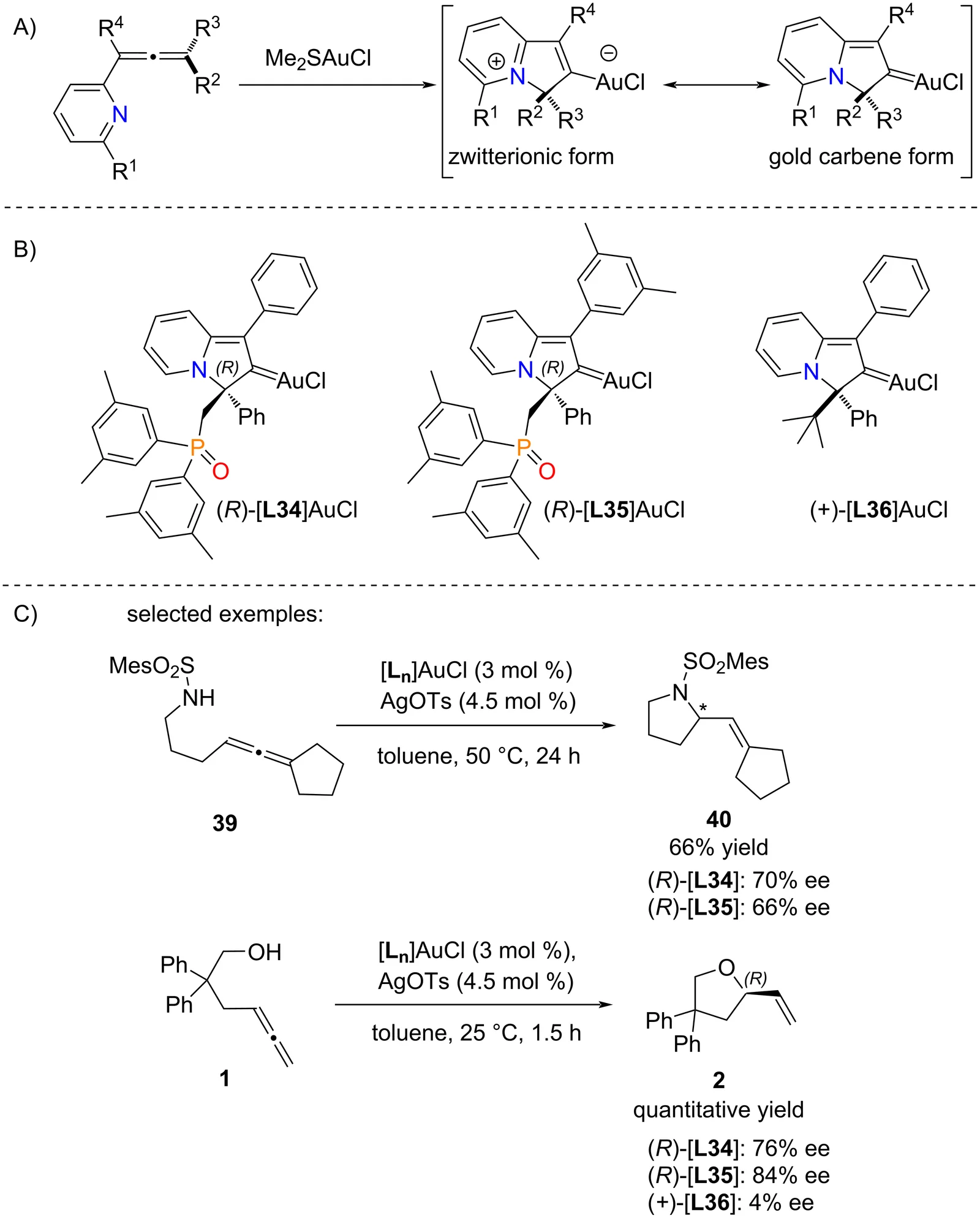
A) Generation of indolizy carbene–gold(I) chloride complexes; B) structure of some indolizy carbene complexes obtained in ref. [81]; C) outcome of the same complexes in cyclization of allene **1** and **39**.

In 2015, our group reported the reactivity of an allene bearing both a phosphine oxide and a pyridine towards gold(I) chloride (Scheme 15) [80]. Due to the activation of the π-system by the metal center, the pyridine ring was able to intramolecularly attack the allene and form a bicyclic compound (Scheme 15A). This resulted in the formation of a new class of coordination compounds named indolizy carbene–gold(I) chloride complexes. In 2021, we then reported the study of the catalytic activity of this family of complexes [81]. These complexes were characterized by having two resonance forms, one with a zwitterionic vinylgold and the other with a gold carbene. Due to the very short Au–C bond distance (1.993(4) Å) measured by SCXRD analysis, reminiscent of other gold carbenes [82–83], this form was described as the more favorable in catalytic applications. Moreover, the sp^3^ carbon atom in between the carbene and the nitrogen atom could be made asymmetric when R^2^ ≠ R^3^. Various chiral gold(I) catalysts were thus synthesized and the enantiomers separated by chiral preparative HPLC (some examples are reported in Scheme 15B as [**L34**]Au(I)Cl, [**L35**]Au(I)Cl, and [**L36**]Au(I)Cl,).

Thus, the intramolecular cyclization of the *N*-mesitylsulfonylallene **39**, yielded the cyclized product **40** in a good yield of 66%, with the best ee of 70% when (*R*)-[**L34**]Au(I)Cl was used. The complex (*R*)-[**L35**]Au(I)Cl showed great activity in the asymmetric cyclization of allenol **1** with excellent yield and very good ee up to 84% for the (*R*) enantiomer (Scheme 15C). The importance of the phosphine oxide moiety was highlighted by the results obtained with (+)-[**L36**]AuCl in which the phosphine oxide was replaced with a *t-*Bu moiety. In fact, although this complex enables quantitative conversion in the hydroalkoxylation of allenol **1**, the enantioselectivity of the reaction decreases markedly to 4%. This observation underscores the crucial role of the phosphine oxide. Accordingly, DFT calculations were performed to elucidate the role of the phosphine oxide moiety.

**Figure 2:**
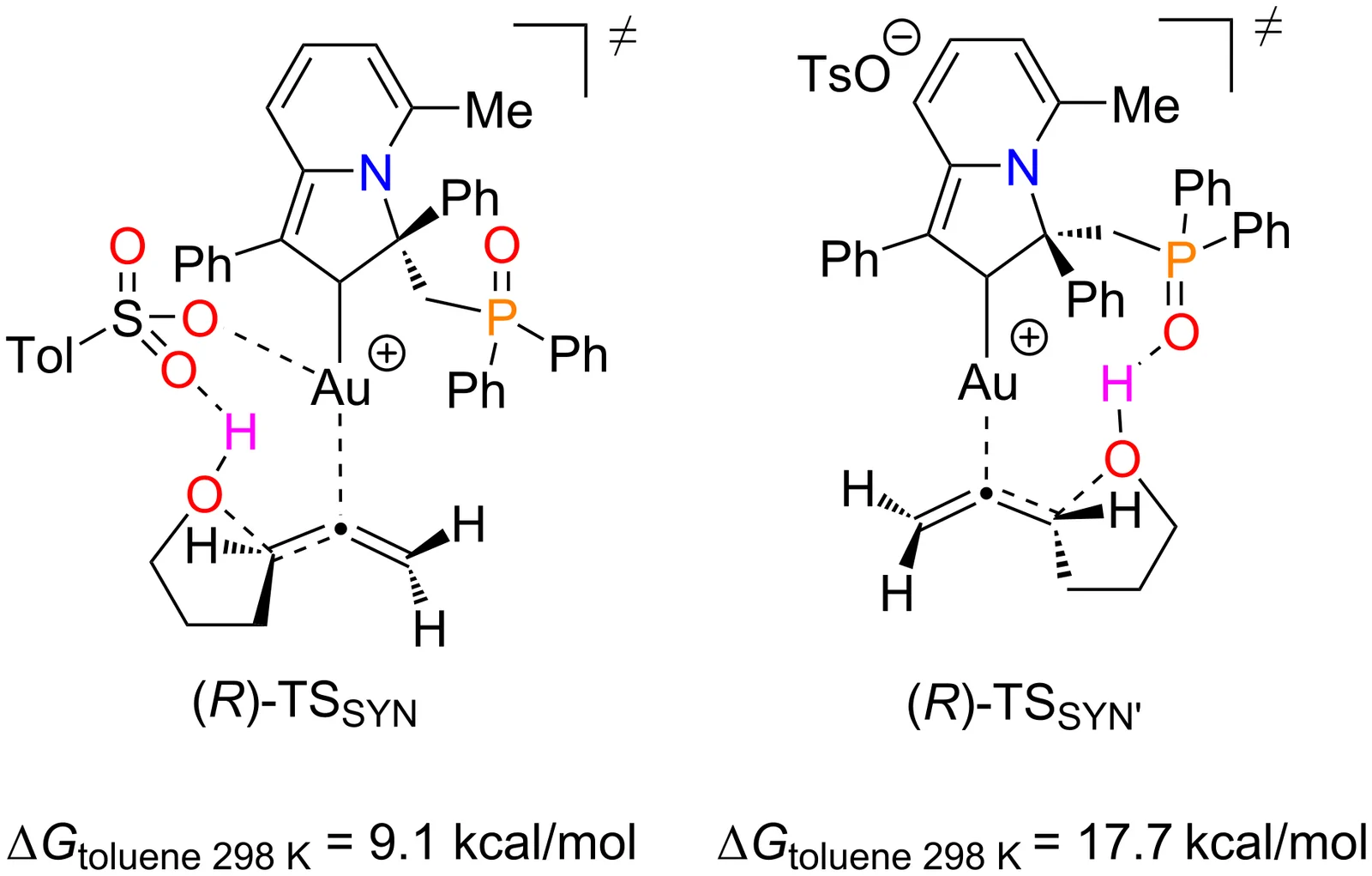
Transition states in the cyclization of hexa-4,5-dien-1-ol by an indolizy carbene–gold(I) complexes showing how the energy barrier depends on the phosphine oxide orientation.

These indolizy ligands showed strong σ-donating properties and high Lewis’s acidity according to spectroscopic data and calculated energy of frontiers orbitals by DFT. The phosphine oxide can act as a Lewis base and may help stabilize cationic gold species or protonated intermediates but not only. DFT studies were performed using hexa-4,5-dien-1-ol as a model to compute the formation of the (*R*)-vinyltetrahydrofuran product. As shown in Figure 2, two transition states leading to the (*R*) enantiomer, differing in the orientation of the phosphine oxide, were modeled. For the lowest-energy transition state, the activation barrier is 9.1 kcal/mol, whereas for the alternative pathway it is 15.5 kcal/mol. The favored pathway corresponds to the orientation in which the oxygen atom of the phosphine oxide is directed toward the nitrogen atom of the indolizine. Owing to the electron-deficient character of the nitrogen atom, the phosphine oxide can interact with it, thereby providing additional stabilization. This interaction locks the conformation of the complex and enables a stereospecific cyclization via an inner-sphere mechanism. In this conformation, steric hindrance from the two aromatic substituents on the phosphorus atom plays a major role in the stereoinduction of the reaction. This is consistent with calculations for the minor enantiomer (*S*), for which the transition state has an activation barrier of 10.4 kcal/mol, thus accounting for the observed enantioselectivity.

**Scheme 16:**
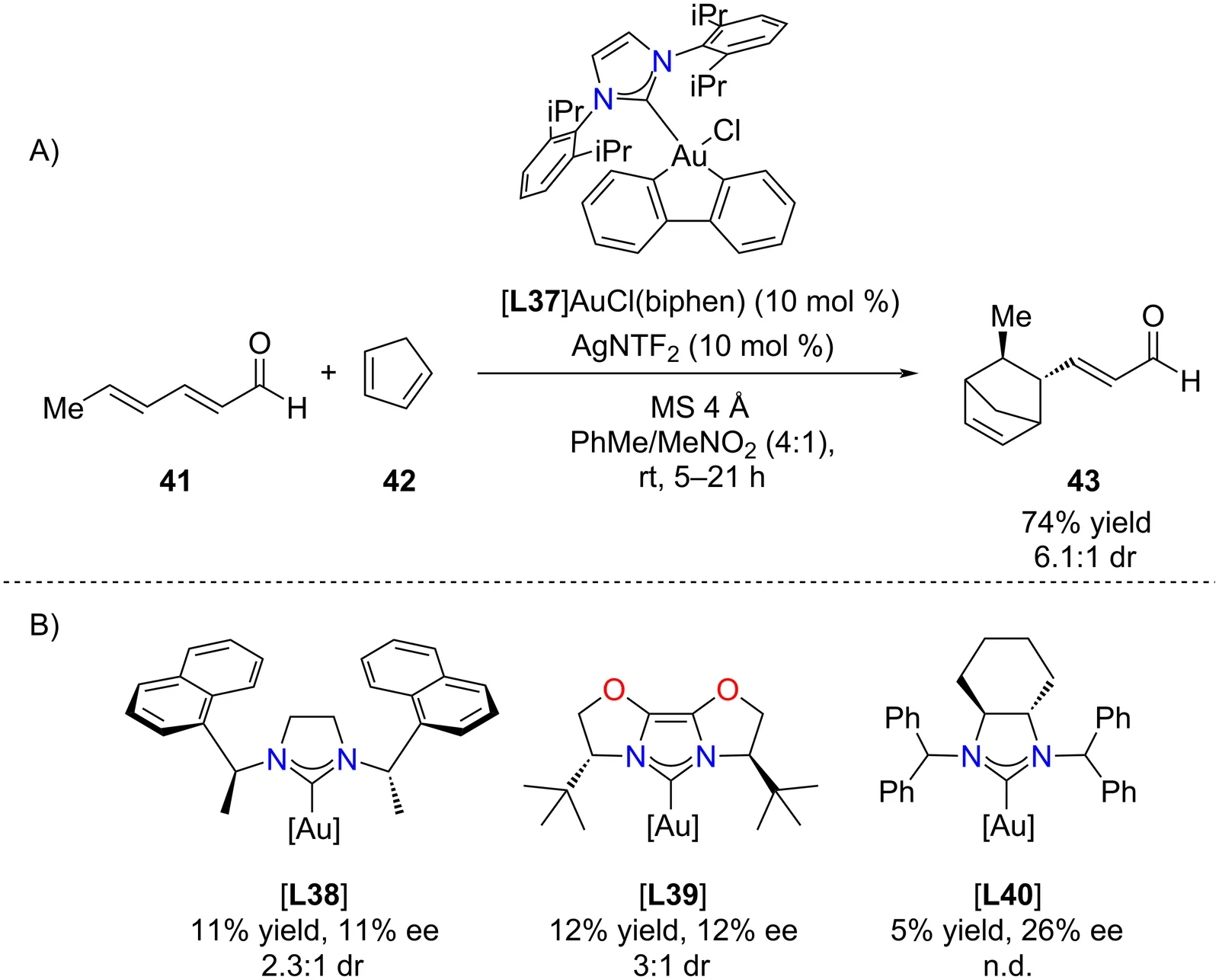
A) Use of NHC–gold(III) complexes in Diels–Alder reaction reported in ref. [84]; B) structure of chiral Au(III) complexes reported in ref. [86].

In 2015, Toste and collaborators reported the synthesis of a stable gold(III) complex by the oxidative addition of IPrAu(I)Cl into the C–C bond of biphenylene to form gold(III) complex [**L37**]Au(III)Cl(biphen) (Scheme 16) [84].

**Figure 3:**
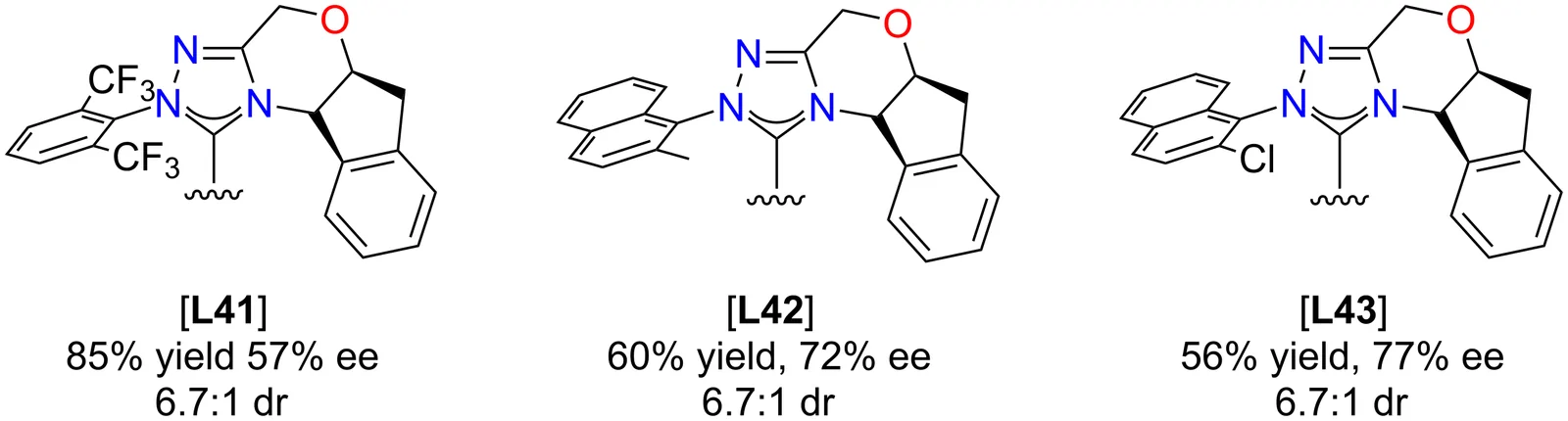
Structure of chiral triazolooxazine-based Au(III) complexes reported in ref. [86] and their outcome in the reaction depicted in Scheme 16A.

**Figure 4:**
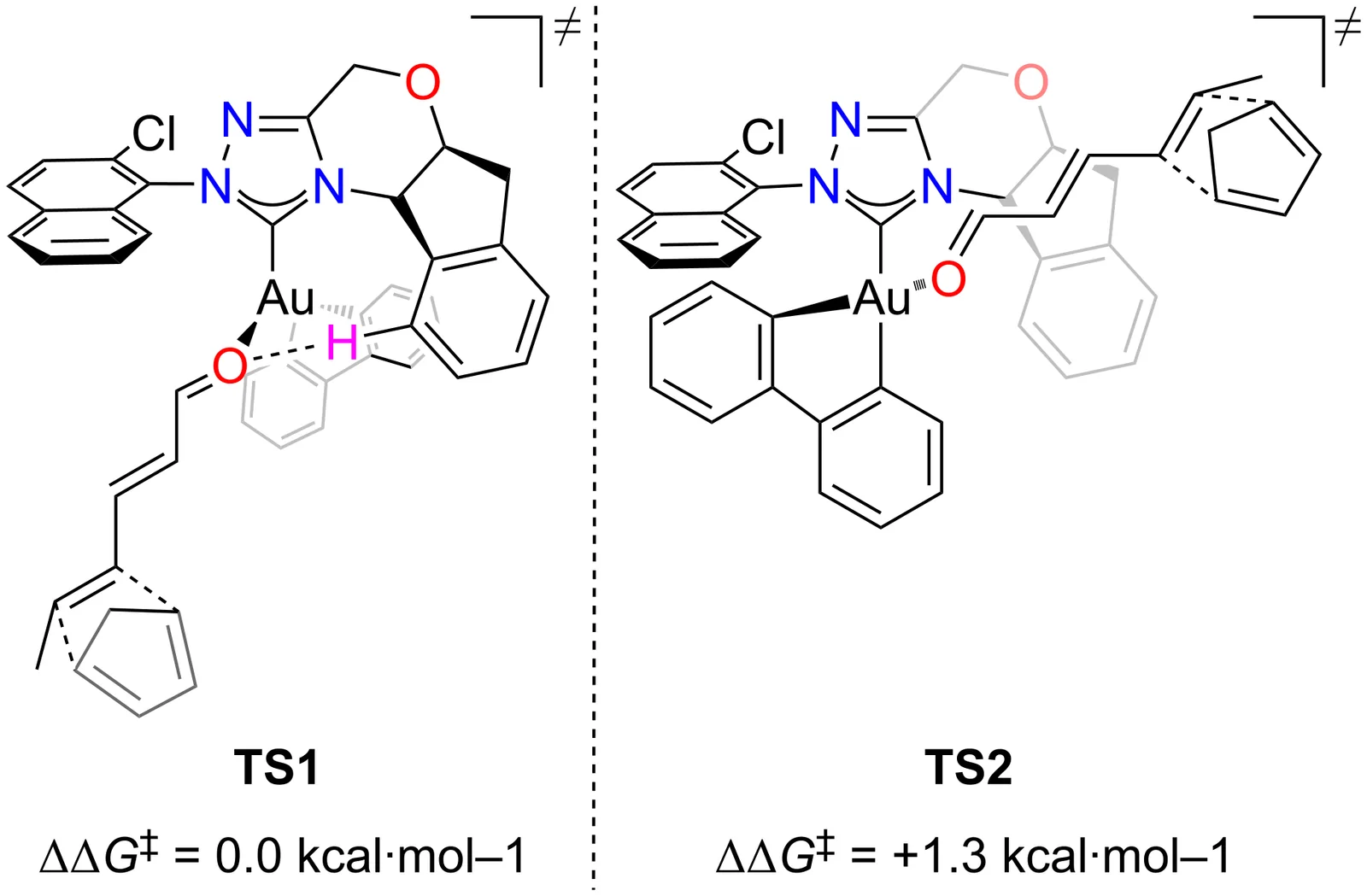
Calculated TS1 (left) and TS2 (right) leading to major and minor enantiomers of the *endo* conformation.

They then studied its reactivity in many reactions, one of them being the Diels–Alder reaction between dienal **41** and cyclopentadiene represented in Scheme 16A. This challenging reaction did not proceed either with the parent gold(I) complexes (IPrAuCl) nor McMillan-type catalysts [85]. On the contrary, [**L37**]Au(III)Cl(biphen) afforded product **43** in 74% yield and good diastereoselectivity. After this seminal paper, in a 2020 study, the same group reported the activity of other chiral gold(III) NHC complexes in the same Diels–Alder reaction [86]. They first examined a series of chiral *C*_2_-imidazolium ligands represented in Scheme 16B. Unfortunately, very low yield and diastereoselectivity were obtained together with very low enantiomeric excess. The authors then shifted their attention from imidazolium- to triazolium-derived ligands. They described 22 new Au(III) catalysts based on triazolium NHC, which are known to be less σ-donating and more π-acidic than “classical” NHCs [87–88]. The most efficient ligands are represented in Figure 3. From their studies, the triazolooxazine core, similar to the one reported by Rovis et al. [89], was the best performing ligand both in terms of yield and enantioselectivity. Indeed, with this core, the yield reached 85% with a good ee of 57% when a 2,6-trifluorophenyl ring was appended on the wingtip (i.e., [**L41**]). When a 2-methylnaphthalene was used in [**L42**], the ee increased to 72%, while when 2-chloronaphthalene was used in [**L43**], the ee reached 77% with a 56% yield. Further studies showed that using trifluorotoluene as solvent and lowering the temperature to 0 °C allowed the former ligand to efficiently catalyze the reaction in 71% yield, 82% ee, and an 8.1:1 dr. In order to explain the origin of the enantioselectivity, the authors calculated the energy of all possible C–C bond formation transition states (TSs) with the [**L43**]Au(III)Cl(biphen) system. The two TSs leading to both enantiomers of the *endo* conformers, TS1 and TS2, are depicted in Figure 4.

**Scheme 17:**
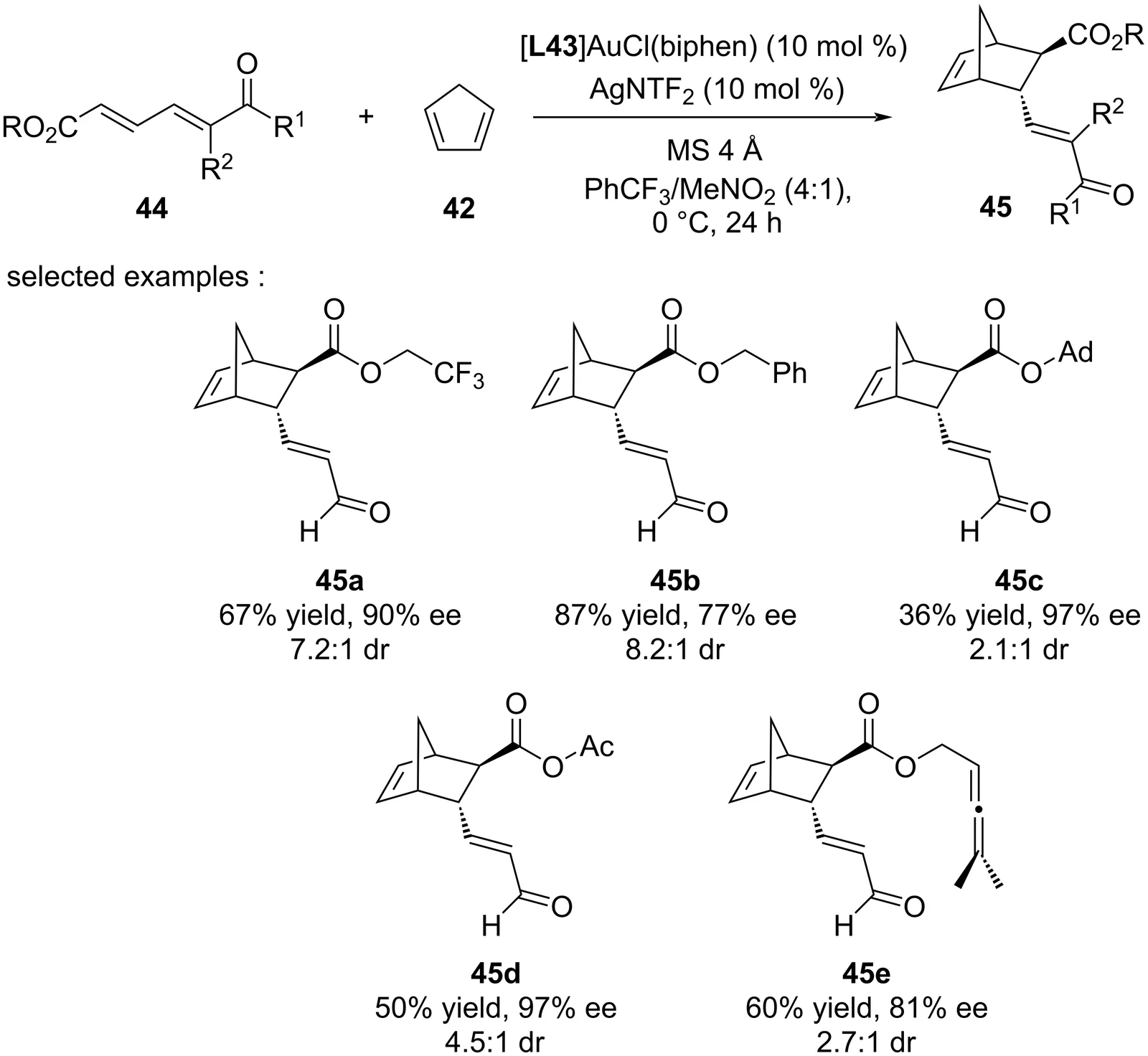
Selected examples of the gold(III)-catalyzed enantioselective Diels–Alder reaction reported in ref. [86].

The main difference between these two transition states is the presence, in TS1, of a favorable interaction between the π-system of the catalyst and the proximal double bond of the substrate, in addition to a C–H···O interaction. This state was 1.3 kcal·mol^−1^ more energetically favored than TS2 which corresponded to a calculated 80% ee, in close agreement with the 77% obtained experimentally. From a series of computational techniques aimed to calculate transition states and multivariate linear regression (MLR) analysis, the authors concluded that two important features for a ligand to be efficient are: (i) a long but not too wide chiral chain, and (ii) the ability to form π–π interactions with the substrate. They also showed that an optimal substrate should not be too long and should have the ability to form various secondary interactions with the ligand and the gold center. From this description, they were able to propose a scope of the bicyclic products with excellent enantiomeric excesses up to 97% and in good yields. Some selected examples are represented in Scheme 17.

## Conclusion

In this review, we have summarized recent advances in the field of asymmetric gold catalysis employing chiral NHC–gold complexes. Although numerous examples have demonstrated excellent enantioselectivity, it is worth noting that a “privileged ligand” for asymmetric gold catalysis is still missing [90]. This is particularly true given the diverse coordination geometries and reactivities of Au(I) and Au(III) complexes. Indeed, the complexes developed so far have found only limited application, being restricted to a small number of benchmark reactions. The studies discussed in this review provide some indications of the structural and experimental factors that may contribute to efficient asymmetric induction, although no general design principle can yet be established.

Regarding ligand design, the examples reported for Au(I), but also for Au(III), suggest that combining a sterically demanding wingtip with a flexible arm bearing the chiral information and a functional group capable of interacting with the substrate through non-covalent interactions may be a promising strategy for achieving higher enantioselectivities. With the exception of a few complexes that exploit axial chirality, central chirality predominates among the catalysts considered here. In the ImPy family, analogous strategies have been employed, featuring a bulky substituent at the C(2) position and a chiral arm bearing a stereogenic center at N(5), or vice versa. In addition, encapsulating the NHC-AuCl moiety within a chiral pocket, such as that provided by cyclodextrins, has also proven to be a good option.

For Au(III) catalysis, the ligand design developed by Toste and co-workers appears particularly interesting. In these systems, the biphenyl group blocks two coordination sites at the metal center, forcing the substrate to bind at a position closer to the chiral ligand. This may provide a more defined spatial relationship between the substrate and the chiral environment than that expected for conventional L–Au(III)-Cl_3_ complexes, for which halide abstraction can generate different species with different possible coordination environments. Whether this approach can be extended to a broader range of Au(III)-catalysed asymmetric reactions remains to be established.

Some reaction conditions also appear to have a substantial influence on the catalytic outcome. No clear preference for a specific solvent can currently be identified, as the optimal solvent varies depending on the reaction investigated. On the other hand, the choice of halide scavenger and, in particular, the nature of the counteranion can significantly affect both reactivity and enantioselectivity. Silver salts bearing weakly coordinating anions (e.g., SbF_6_^−^ or PF_6_^−^) have been successfully employed, although some studies suggest that more coordinating counteranions, like OTs^−^, OTf^−^, NTf_2_^−^, may favor higher enantioselectivities.

From a ligand-design perspective, several strategies could be explored in future studies. One possibility would be the development of NHC ligands bearing two chiral wingtips, so that both sides of the ligand can contribute to the chiral environment.

An interesting approach involving the use of prochiral NHC ligand has been developed by Clavier and collaborators in order to synthesized optically pure (>98% ee) *C*_1_- and *C*_2_-symmetric NHC–transition metal complexes with axial chirality. The stable atropisomers were efficiently resolved by chiral preparative HPLC. First-ever, carbene transfer from an optically pure Cu complex to Au or Pd was shown to occur with complete stereoretention [91].

Whether these alternative modes of chirality can translate into more effective stereocontrol in gold catalysis remains an open question, but they may offer new opportunities for controlling the three-dimensional environment around the gold center. We hope that this review will serve as a useful reference for researchers working in this field and stimulate further ideas and developments towards the design of more efficient chiral NHC–gold catalysts for asymmetric catalysis.

## Data Availability

Data sharing is not applicable as no new data was generated or analyzed in this study.
